# Gut oralisation signatures in Parkinson's disease: an integrative multi-study analysis

**DOI:** 10.1080/20002297.2026.2737700

**Published:** 2026-10-11

**Authors:** Gabriela Durão, Emile Boyer, Martine Bonnaure-Mallet, Marc Verin, Manon Auffret, Vincent Meuric

**Affiliations:** a INSERM, INRAE, UMR 1317, Nutrition, Metabolisms and Cancer (NuMeCan) Institute, Université de Rennes, Rennes, France; b Brain-Clinicial and Experimental Neuroplasticity (B-CLINE), Laboratoire Interdisciplinaire pour l'Innovation et la Recherche en Santé d'Orléans (LI2RSO), Université d'Orléans, Orléans, France; c Neurology Department, CHU d'Orléans, Orléans, France; d France Développement Electronique (FDE), Monswiller, France; e CIC 1414, CHU de Rennes - Université de Rennes, Rennes, France

**Keywords:** Parkinson's disease, gut microbiome, 16S rRNA gene sequencing, meta-analysis, oralisation, eHOMD, *Streptococcus mutans*, oral health, caries-associated bacteria, periodontal bacteria

## Abstract

**Background:**

Parkinson’s disease (PD) compromises oral hygiene and oral health, potentially increasing oral bacterial input to the gut. Whether PD is associated with reproducible faecal enrichment of oral disease–linked taxa remains unclear.

**Objective:**

To determine whether oral disease–linked taxa are enriched in the faecal microbiota of individuals with PD.

**Methods:**

We conducted a pooled re-analysis of 23 faecal 16S rRNA datasets, comprising 3,594 PD and control samples. eHOMD was used to resolve oral-associated taxa, including caries/endodontic- and periodontitis-linked taxa. SILVA-based profiles were used to assess community diversity and ordination patterns. Differential-abundance analyses accounted for variation between studies.

**Results:**

PD samples showed heterogeneous but reproducible enrichment of oral disease–linked taxa, with a prominent caries/endodontic-associated component. The cariogenic-enriched subgroup showed reduced evenness and increased representation of oral taxa including *Streptococcus mutans*. Beta-diversity analyses showed substantial PD-control overlap and an oralization-related compositional gradient rather than a discrete PD-specific cluster.

**Conclusions:**

This pooled re-analysis identifies a gut oralization-associated signal in a subset of individuals with PD. Faecal enrichment of oral-associated taxa may represent one component of gut microbiome heterogeneity and may indirectly reflect variation in oral ecosystem status, supporting oral health assessment in future PD microbiome studies.

## Key messages

- This pooled re-analysis of 23 public faecal 16S rRNA datasets from 21 BioProjects identifies a reproducible gut oralisation-associated signal in a substantial subset of individuals with Parkinson's disease.
- PD-associated oralisation was heterogeneous and organised along an oralisation-related compositional gradient, with a prominent caries/endodontic-associated component and no generalised periodontitis-associated pattern.
- These findings support the integration of oral health assessment, dental/periodontal phenotyping, diet and detailed clinical metadata into future PD microbiome studies.

## Introduction

Oral health impairment is common in Parkinson's disease (PD) and remains insufficiently addressed in routine care [1,2]. Motor symptoms, including tremor, bradykinesia and rigidity, together with non-motor features such as dysphagia, xerostomia, cognitive impairment and affective symptoms, can compromise daily oral hygiene, reduce access to regular dental care and limit adherence to preventive measures. Accordingly, people with PD often show greater plaque accumulation and a higher burden of dental caries, periodontal disease, tooth loss and prosthetic complications than controls, although estimates vary across studies [1,2].

At the same time, growing evidence supports the existence of an oral–gut axis, whereby oral bacteria can reach the gastrointestinal tract and become over-represented in stool under specific host, ecological and treatment-related conditions [3]. The detection of oral-associated taxa in stool is often interpreted as an ecological footprint compatible with oral dysbiosis, although recent work suggests that it may also reflect depletion of the resident gut microbiota and reduced colonisation resistance [4]. In PD, several studies have reported increased faecal representation of taxa typically associated with the oral cavity, including lactic-acid-producing bacteria and cariogenic taxa such as *Streptococcus mutans* [5]. These observations are particularly relevant because oral diseases are common in PD and because experimental findings suggest that oral-associated taxa may contribute to PD-relevant metabolic and pathological processes [6].

Recent large-scale meta-analyses have established that PD is associated with a reproducible yet heterogeneous faecal microbiome signature across cohorts [7]. However, these studies were not designed to determine whether part of this recurrent signal reflects a structured enrichment of oral disease-associated bacteria in the gut. This question is biologically and clinically relevant. From a biological perspective, faecal detection of oral taxa may indicate ectopic transfer of oral bacteria rather than stable intestinal colonisation [8]. From a clinical perspective, if reproducible, such an ‘oralisation’ signal could provide insight into PD heterogeneity and highlight oral health as an underappreciated dimension of disease management [1].

Here, gut oralisation in PD was investigated through a large-scale, harmonised re-analysis of 23 publicly available faecal 16S rRNA gene datasets from 21 BioProjects comprising 2,154 PD and 1,440 control samples across 21 publications. Gut oralisation was operationally defined as the detection and enrichment of oral-associated bacteria in faecal microbiota. Using the expanded human oral microbiome database (eHOMD) to improve the detection of oral-associated taxa, the enrichment of caries/endodontic- and periodontitis-associated taxa was quantified in PD stools compared with controls. SILVA-based analyses were then used to determine whether these exploratory oral-enrichment profiles were associated with broader gut community structure. By focusing on oral disease-associated taxa detected in faecal samples, this study examines whether gut oralisation represents a reproducible component of PD-associated gut microbiome heterogeneity and a potential indirect marker of variation in oral ecosystem status.

## Methods

The main methodological steps are summarised below, while detailed dataset-specific preprocessing parameters, taxonomic assignment procedures and statistical analyses are provided in the Supplementary Methods and Supplementary Tables.

### Study identification and inclusion criteria

A systematic search was conducted for publicly available human faecal 16S rRNA gene datasets comparing PD and control samples, with raw sequencing data deposited in international repositories, including NCBI SRA and ENA. PubMed was searched with no year restriction using a broad, sensitivity-oriented query (last accessed 30 September 2025; full query provided in the Supplementary Methods). Inclusion criteria were: (i) human participants; (ii) stool/faecal 16S rRNA amplicon sequencing; (iii) available PD/control status and (iv) accessible raw sequencing reads with valid repository identifiers. Exclusion criteria included animal studies, non-stool sampling, reviews, systematic reviews or meta-analyses, shotgun-only studies and inaccessible raw sequencing data or invalid repository identifiers. Only samples classified as either PD or controls were retained; samples from prodromal PD, essential tremor or other non-eligible clinical categories were excluded. For longitudinal datasets, only baseline samples were included, and follow-up samples or replicates were removed so that each participant contributed a single exploitable faecal sample.

### Sequence retrieval and processing (QIIME 2)

Raw FASTQ files were retrieved from SRA/ENA using accession numbers extracted from eligible publications (Table S1). Because this study is a harmonised reanalysis of publicly available cohorts, sequencing design and raw-read quality were inherited from the original studies. Datasets were processed independently by dataset and 16S rRNA gene region in QIIME 2 v2025.10 [9]. Study-specific primers and adaptors were removed with Cutadapt, and ASVs were inferred with DADA2 after quality filtering, denoising, chimera removal and dataset-specific read truncation (Table S2) [10]. Samples with fewer than 5,000 reads after denoising were excluded. When paired-end reads could not be merged because quality-based truncation during our reprocessing left insufficient overlap, datasets were processed using forward reads only and the resulting single-end ASV tables were used for downstream analyses (Table S2).

### Taxonomic profiling with eHOMD and SILVA

Taxonomy was assigned with q2-feature-classifier using region-specific naïve Bayes classifiers trained on two complementary reference databases. eHOMD v16.01 was used to refine the annotation of oral-associated taxa and quantify gut oralisation signatures [11,12], whereas SILVA release 138 SSURef NR99 was used for broader community-level profiling, including alpha-diversity, beta-diversity and ordination analyses [13].

### Statistical analyses and oral-enrichment profiling

All statistical analyses were performed in R version 4.5.1 within RStudio. Taxonomic count and relative-abundance tables were generated from ASV tables aggregated at the lowest resolved taxonomic level, when taxonomic assignment allowed. Differential abundance analyses were performed using two complementary approaches, MaAsLin2 and ANCOM-BC2 [14,15]. Taxonomic count tables were generated independently for each study and subsequently merged for pooled modelling. In both frameworks, case–control status (PD vs Controls) was modelled as the primary variable of interest, with amplified 16S rRNA gene region included as a fixed effect and BioProject ID included as a random intercept to account for between-study heterogeneity. Low-prevalence taxa were removed using a 10% prevalence threshold. In MaAsLin2, count tables were normalised by total-sum scaling and log-transformed before linear modelling. ANCOM-BC2 was applied to the corresponding pooled count table as a complementary compositional differential-abundance approach accounting for sampling-fraction bias. *P*-values were adjusted using the Benjamini–Hochberg false discovery rate procedure, with q < 0.05 considered statistically significant. A MaAsLin2-based sensitivity analysis was performed to assess case–control status and amplicon-region associations for oral disease-linked taxa; full classification criteria are provided in the Supplementary Methods.

To assess robustness to read-processing strategy, MaAsLin2 and ANCOM-BC2 were rerun after excluding the four originally paired-end datasets requiring forward-only processing and, in a more restrictive analysis, using only datasets successfully processed as paired-end. PD-versus-control effect estimates were compared with those from the primary analysis using Spearman rank correlations, and effect-direction concordance was assessed for taxa significant in the primary analysis (Supplementary Methods).

For the original exploratory oral-enrichment profiles, oral disease-associated enrichment was assessed by annotating taxa detected in the MaAsLin2 and ANCOM-BC2 differential-abundance outputs using two predefined categories derived from eHOMD annotations and the literature: caries/endodontic-associated taxa and periodontitis/subgingival plaque-associated taxa. For each method, taxa assigned to either category and associated with case–control status were retained for score construction using a prespecified exploratory threshold of q < 0.05. These scores were used as exploratory summaries of disease-associated oral taxa rather than as diagnostic classifiers. Sample-level caries/endodontic and periodontitis scores were calculated as the cumulative relative abundance of retained taxa within each category. Method-specific enrichment thresholds were defined as the mean score among controls. The mean control score was used as a pragmatic background threshold because it provides a conservative cutoff in skewed control distributions, reducing the likelihood of classifying samples with only marginal oral-taxon signals as enriched. Samples were then classified into four mutually exclusive profiles according to whether their caries/endodontic and/or periodontitis scores exceeded the corresponding control-derived threshold: no oral enrichment, caries/endodontic enrichment, periodontitis enrichment or combined enrichment. Differences in oral-enrichment profile distributions between controls and PD samples were assessed using 2 × 4 contingency tables. Pearson's χ^2^ tests were used for global comparisons, followed by profile-wise Fisher's exact tests comparing each profile against all remaining profiles. *P*-values were adjusted using the Benjamini–Hochberg procedure. Additional sensitivity analyses addressing potential circularity and assessing the robustness of oral-profile construction are described in the Supplementary Methods.

As a complementary continuous analysis, a cariogenic-to-periodontitis balance was calculated for each sample as the scaled log-ratio difference between retained caries/endodontic-associated taxa and periodontitis-associated taxa, after addition of a 1 × 10⁻^6^ pseudo-count. Positive values indicate a shift toward caries/endodontic-associated taxa, whereas negative values indicate a shift toward periodontitis-associated taxa. Balance distributions were compared between controls and PD samples using Wilcoxon rank-sum tests, followed by Benjamini–Hochberg correction across methods.

Alpha and beta diversity were assessed using SILVA-derived taxonomic profiles aggregated at the lowest reliably resolved taxonomic level. For alpha diversity, observed richness, Shannon diversity and Pielou's evenness were calculated. Differences between PD and control samples were tested globally and within each oral-enrichment profile using Wilcoxon rank-sum tests, with Benjamini–Hochberg correction.

For beta diversity, Bray–Curtis dissimilarities were calculated from relative-abundance profiles, whereas Aitchison distances were calculated from count profiles after compositional processing, including a 1% prevalence filter, CZM zero replacement and centred log-ratio transformation. Principal coordinates analysis (PCoA) was used to visualise unconstrained community variation. Associations between community composition and case–control status were tested using marginal PERMANOVA, with BioProject included as a model term and permutations constrained within BioProject. The primary analysis included all 21 BioProjects, with a sensitivity analysis restricted to the 18 BioProjects containing both PD and Controls, thereby excluding the three BioProjects containing PD samples only. Multivariate dispersion between PD and Controls was assessed using betadisper. Distance-based redundancy analysis was then used to descriptively visualise variation associated with case‒control status and the exploratory oral-enrichment profiles after conditioning on BioProject.

To assess potential circularity in these profiles, beta-diversity analyses were repeated after removing all taxa used to define the original profiles and recalculating Bray–Curtis and Aitchison distances; full details are provided in the Supplementary Methods.

## Results

### Study selection and dataset characteristics

The study selection process is summarised in Figure 1 using a PRISMA 2020 flow diagram. Overall, 21 studies met the inclusion criteria and contributed 23 datasets from 21 BioProjects that were processed using both eHOMD and SILVA reference databases. The final dataset included 3,594 faecal samples, comprising 1,440 controls and 2,154 confirmed PD samples (Table S1).

**Figure 1.**
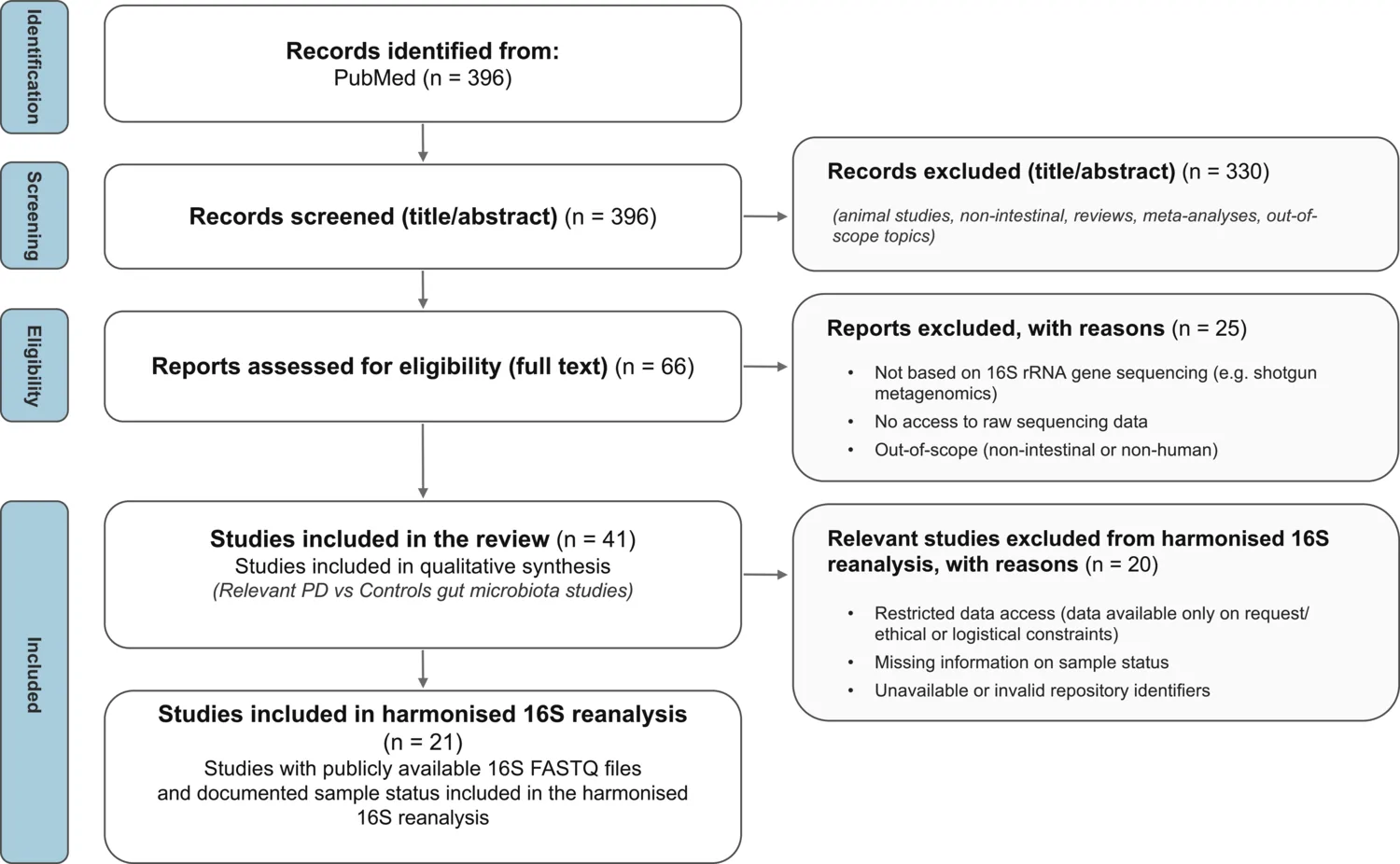
PRISMA 2020 flow diagram of study selection. Of 396 records identified through PubMed, 41 studies were retained for qualitative synthesis, and 21 studies with publicly available raw 16S rRNA sequencing data and documented sample-level PD/control status were included in the harmonised reanalysis.

### Differential abundance of oral disease-linked taxa (eHOMD)

MaAsLin2 and ANCOM-BC2 identified partially overlapping oral disease-linked taxa associated with PD (Table S3). Among caries/endodontic-associated taxa, both methods consistently showed enrichment of *Bifidobacterium* and *Lactobacillus*, together with depletion of *Veillonella* and *Veillonella atypica* (Fig. S1). MaAsLin2 additionally identified a broader set of enriched caries/endodontic-associated taxa, including *Streptococcus mutans*, *Eggerthia catenaformis*, *Streptococcus anginosus*, *Bifidobacterium dentium*, *Finegoldia magna* and *Pseudoramibacter alactolyticus* (Table S3; Fig. S2). In contrast, ANCOM-BC2 supported fewer significant caries/endodontic enrichments, but additionally detected depletion of *Veillonella parvula* (Table S3; Fig. S2). Periodontitis/subgingival plaque-associated taxa showed more method-dependent associations. *Methanobrevibacter oralis* was enriched, whereas *Lachnoanaerobaculum* was depleted in PD samples across both methods. Additional periodontitis-associated taxa were enriched mainly in MaAsLin2, whereas ANCOM-BC2 identified depletion of *Dialister invisus*, *Rothia mucilaginosa*, *Lautropia dentalis* and *Alloprevotella* taxa (Table S3; Fig. S2).

A MaAsLin2-based sensitivity analysis including both case‒control status and 16S rRNA gene target region in the model indicated that most oral disease-linked taxa were classified as PD-associated, whereas a subset showed mixed PD/region-associated or region-associated signals (Table S5). Mixed patterns were observed for several caries/endodontic-associated taxa, including *Bifidobacterium dentium*, *Bifidobacterium longum* and *Finegoldia magna* and for periodontitis/subgingival plaque-associated taxa such as *Tannerella* and *Selenomonas* sp. HMT-442. These results indicate that amplicon-region effects were taxon-specific and affected a subset of the oral disease-linked taxa, while most retained significant associations with case–control status.

PD-versus-control effect estimates were robust in read-processing sensitivity analyses. Exclusion of the four datasets requiring forward-only processing produced highly concordant effects relative to the full analysis (Spearman *ρ* = 0.97 for both MaAsLin2 and ANCOM-BC2), and restriction to successfully paired-merged datasets showed similarly high concordance (*ρ* = 0.95 and 0.97, respectively; Table S4, Fig. S3). Among taxa significant in the full analysis and retained in the corresponding sensitivity models, all preserved the same direction of effect across both sensitivity analyses.

### Oral-enrichment groups

PD samples showed significantly different oral-enrichment profile distributions compared with controls for both MaAsLin2- and ANCOM-BC2-derived taxa sets (Figure 2A). In MaAsLin2-derived profiles, enrichment for at least one oral disease-linked taxa set was observed in 62.5% of PD samples compared with 53.2% of controls, with the largest shift corresponding to an increased cariogenic profile in PD (31.1 vs 19.1%). ANCOM-BC2-derived profiles showed a concordant increase in the cariogenic profile among PD samples (26.6 vs 15.1%). In contrast, periodontitis-only profiles were more frequent in controls in both analyses. Global 2 × 4 contingency-table tests confirmed significant differences in profile distributions for both MaAsLin2 (χ^2^ = 92.56, BH-adjusted *p* < 0.0001, Cramér's V = 0.160) and ANCOM-BC2 (χ^2^ = 81.49, BH-adjusted *p* < 0.0001, Cramér's V = 0.151). Post-hoc Fisher tests identified the cariogenic profile as the most reproducible PD-associated enrichment pattern across methods.

**Figure 2.**
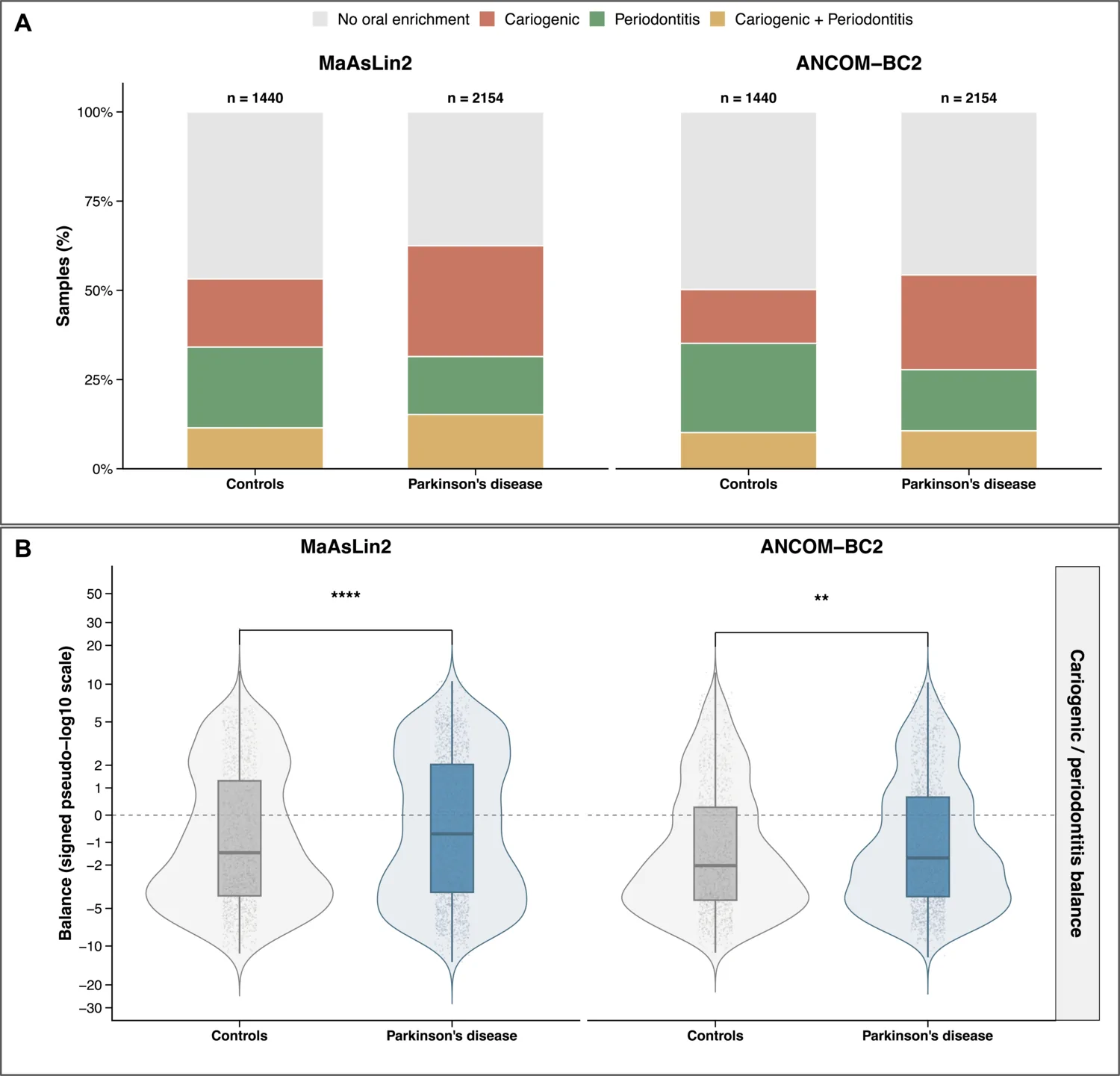
Oral disease-associated enrichment profiles in PD and controls. (A) Proportion of stool samples classified as no oral enrichment, cariogenic enrichment, periodontitis enrichment, or combined cariogenic + periodontitis enrichment, shown separately for MaAsLin2- and ANCOM-BC2-derived taxa sets. (B) Distribution of the cariogenic-to-periodontitis balance in controls and PD samples. Higher values indicate a shift toward caries/endodontic-associated taxa. Differences in balance between PD and controls were assessed using two-sided Wilcoxon rank-sum tests. **P < 0.01; ****P < 0.0001.

As a complementary continuous measure, the cariogenic-to-periodontitis balance was compared between PD and control samples (Figure 2B). PD samples showed significantly higher balance values than controls for both MaAsLin2- and ANCOM-BC2-derived taxa sets, consistent with the increased frequency of the cariogenic profile in the categorical analysis.

As an independent sensitivity analysis addressing potential circularity in taxon selection, a literature-derived caries/endodontic panel also showed higher relative representation in PD, whereas no significant association was observed for the literature-derived periodontitis panel (Table S6), providing independent support for the caries/endodontic-associated enrichment observed in the primary analysis.

### Alpha diversity across oral-enrichment categories

Using SILVA-derived profiles, alpha diversity did not differ significantly between PD and control samples overall after FDR correction (Figure 3). Stratified analyses across oral-enrichment profiles showed no significant group differences in observed richness, Shannon diversity or Pielou's evenness within the no-enrichment, periodontitis-enriched or combined-enrichment subgroups. The only significant difference was observed within the cariogenic-enriched subgroup, where Pielou's evenness was lower in PD samples than in controls (median 0.698 vs 0.712; *p* = 0.0054; FDR = 0.0272) (Figure 3C), while richness and Shannon diversity remained comparable.

**Figure 3.**
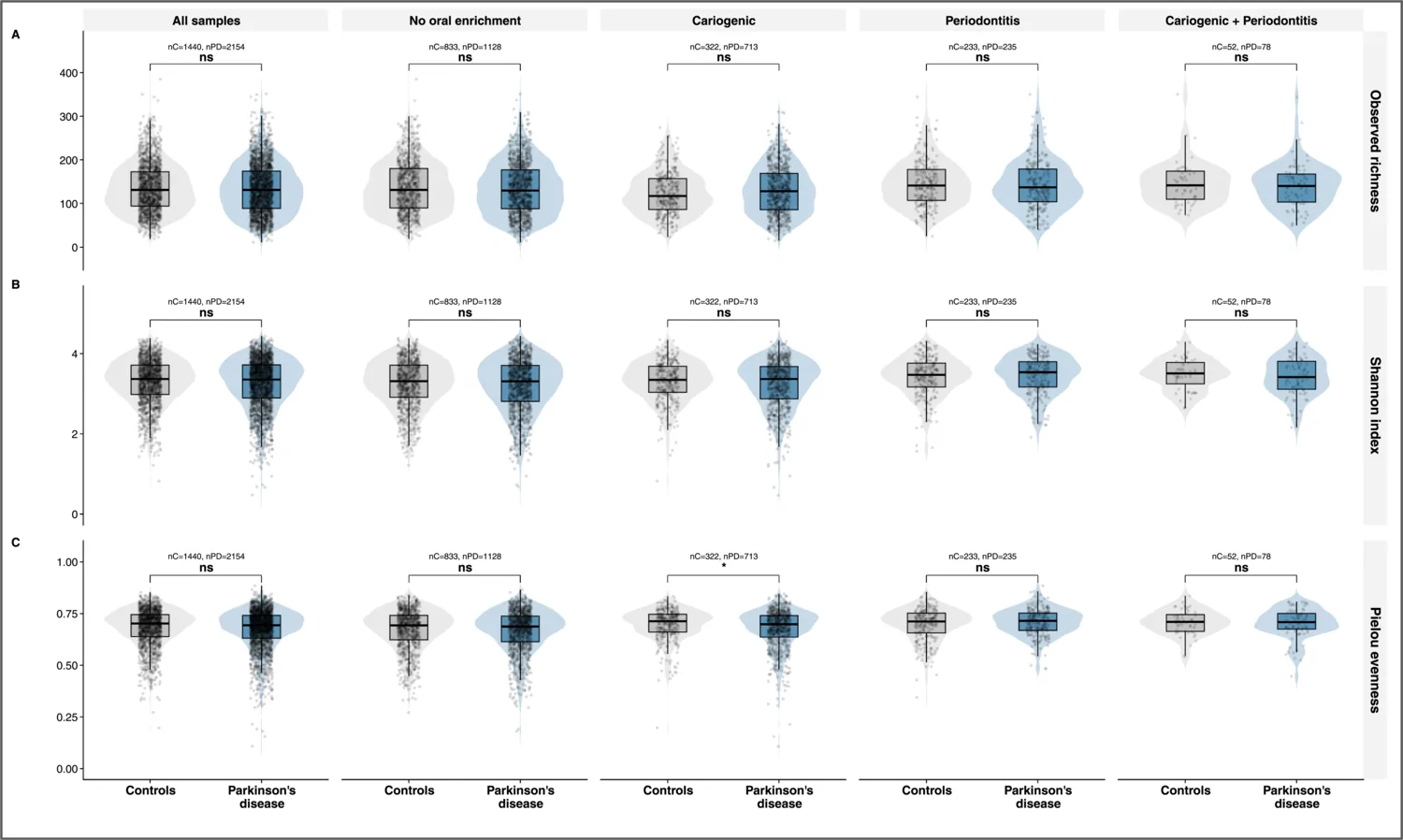
Alpha diversity across oral-enrichment categories in PD and controls. (A) Observed richness, (B) Shannon diversity and (C) Pielou's evenness are shown for all samples and for no oral enrichment, cariogenic enrichment, periodontitis enrichment or combined cariogenic + periodontitis enrichment subgroups. *P < 0.05; ns, not significant.

### Beta diversity and constrained ordination

After accounting for BioProject structure, case–control status was associated with a small but statistically significant proportion of variation in gut microbial community composition. In the primary analysis including all 21 BioProjects (*n* = 3,594), case–control status accounted for 0.43% of Bray–Curtis variation (R^2^ = 0.00432, F = 18.86, *p* = 0.001) and 0.22% of Aitchison variation (R^2^ = 0.00217, F = 10.28, *p* = 0.001). By comparison, BioProject accounted for 17.43% of Bray–Curtis and 24.33% of Aitchison variation. Results were similar when the analysis was restricted to the 18 BioProjects containing both PD and Controls (Bray–Curtis R^2^ = 0.00479; Aitchison R^2^ = 0.00230), indicating that the three PD-only BioProjects did not materially affect the observed association (Table S7A).

Multivariate dispersion also differed between PD and Controls in the 18 mixed BioProjects, with PD samples showing slightly greater mean distances to the group spatial median than Controls for both Bray–Curtis (0.568 vs 0.548; F = 79.36, *p* = 0.001) and Aitchison distances (34.73 vs 33.19; F = 92.83, *p* = 0.001). Given these dispersion differences, the PERMANOVA results were interpreted cautiously as a statistically significant association with a modest effect size, rather than as evidence of a clear separation between PD and Control microbial communities (Table S7B).

Consistent with the small PERMANOVA effect size of case–control status, unconstrained PCoA plots showed extensive overlap between PD and Control samples for both Bray–Curtis and Aitchison distances (Fig. S4 A and C), whereas samples showed more apparent organisation according to the exploratory oral-enrichment profiles (Fig. S4 B and D). Project-conditioned dbRDA ordinations were consistent with an oralisation-related compositional gradient, with substantial overlap between PD and control samples. Oral-profile centroids projected in different directions within the constrained ordination space, particularly for the cariogenic and periodontitis-associated profiles (Figure 4). Similar profile-related patterns were observed when the control and PD samples were visualised separately (Fig. S5). In a sensitivity analysis excluding all taxa used to define the original oral-enrichment profiles before recalculating beta-diversity, profile-related structure was attenuated, particularly in the Bray–Curtis ordination. Nevertheless, profile-related organisation remained visible with both distance metrics (Fig. S6; Supplementary Results).

**Figure 4.**
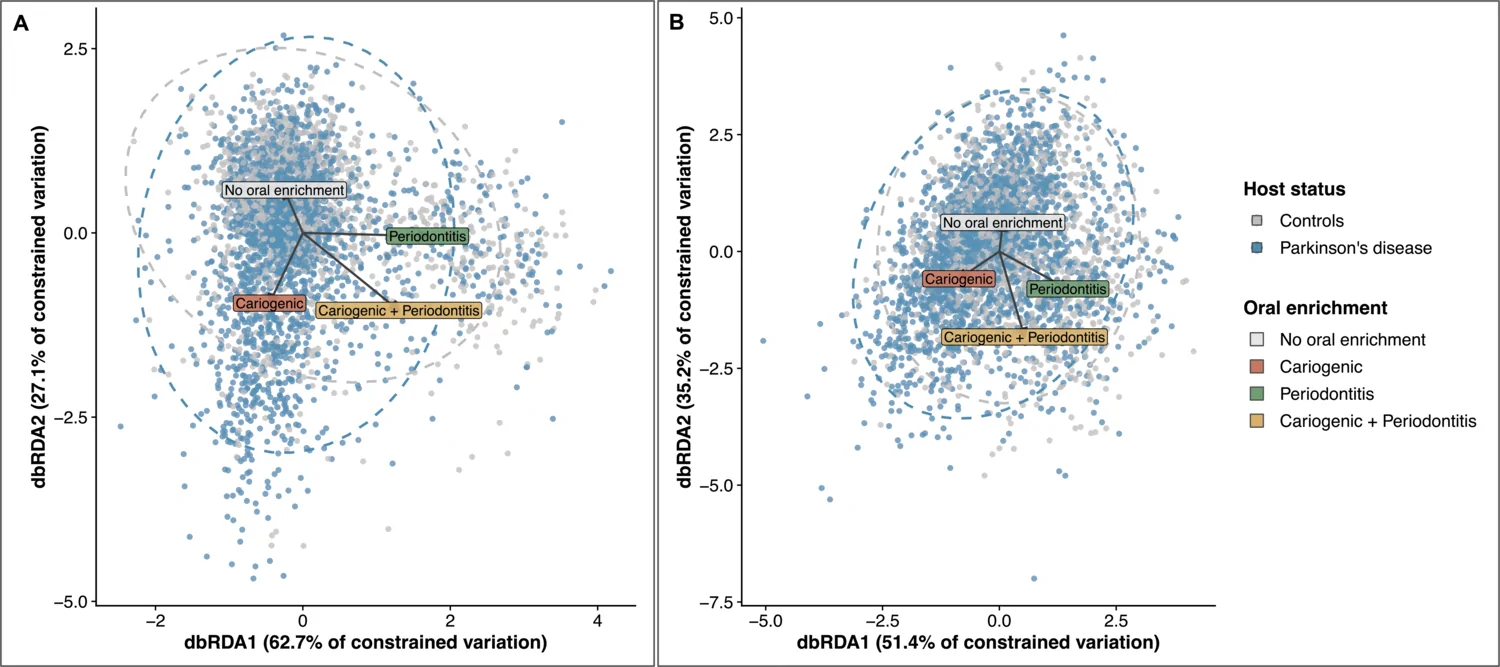
dbRDA of gut microbiome beta diversity in PD and controls using Bray–Curtis (A) and Aitchison (B) distances. Samples are coloured by case‒control status, and arrows indicate oral-enrichment category centroids in the constrained ordination space after conditioning on BioProject. Axis percentages indicate the proportion of constrained variation represented by each dbRDA axis.

## Discussion

### Principal findings and context

This re-analysis of nearly 3,600 faecal samples from 23 public datasets across 21 BioProjects, combining eHOMD- and SILVA-based taxonomic profiling, supports the presence of a reproducible gut oralisation signal in PD, characterised by enrichment of oral disease-associated taxa in a subset of individuals. Rather than defining a uniform disease-specific microbiome state, this signal was heterogeneous across patients and appeared to organise along an oralisation-related compositional gradient, with caries/endodontic-associated taxa contributing prominently to this oralisation pattern. Importantly, the caries/endodontic-associated component was also observed using an external literature-derived panel defined independently of PD association testing, indicating that this pattern was not solely driven by the original taxon-selection strategy. Although global alpha diversity was largely preserved, the cariogenic-enriched subgroup showed reduced community evenness. This may reflect a more pronounced compositional imbalance in this subset, in which the relative expansion of a limited number of taxa could reduce the even distribution of the remaining community. Given the enrichment of several caries/endodontic-associated taxa observed in these samples, oral-associated taxa may form part of this shift, although their direct contribution to the reduced evenness cannot be established from the present analysis. Together, these findings indicate that a subset of individuals with PD shows a reproducible cariogenic-associated gut oralisation pattern within a broader heterogeneous microbial landscape, rather than reflecting a single homogeneous microbial configuration.

### Comparison with prior work

Previous cohort studies and meta-analyses have consistently reported gut microbiota alterations in PD, including enrichment of opportunistic taxa and depletion of short-chain fatty acid-producing taxa [16,17]. More recent large-scale analyses further indicate that the PD-associated faecal microbiome signal is reproducible across cohorts but highly heterogeneous. For example, in a large-scale multi-cohort re-analysis, Romano et al. [7] found that disease status explained ≤1% of microbiome variation, whereas study of origin accounted for 19.9% of the variation in 16S datasets [7]. However, these studies did not specifically assess whether oral disease-associated bacteria contribute to this heterogeneity. Our results suggest that enrichment of oral-associated taxa in the gut may represent one component of the inter-individual and inter-cohort variability observed in PD.

Here, multiple oral disease-linked or oral-associated taxa were differentially associated with PD, including cariogenic/endodontic-associated taxa such as *Streptococcus mutans*, *Streptococcus anginosus* and *Bifidobacterium longum* [18,19]. This is consistent with a shotgun metagenomic study that reported increased faecal representation of oral-associated bacteria in PD, including prominent signals for cariogenic taxa such as *S. mutans* [5]. The recurrence of these taxa across multiple datasets and analytical approaches supports a reproducible faecal signal involving oral-associated taxa in PD.

At the community level, the absence of a robust global reduction in alpha diversity is also consistent with recent meta-analytic findings [20]. However, oralisation-based stratification revealed ecological differences that were not apparent in the overall PD-control comparison. This suggests that some microbiome differences in PD may be concentrated within specific subgroups and therefore be less apparent in analyses treating PD as a single homogeneous group.

### Interpretation and biological plausibility

Because this study relied on cross-sectional 16S rRNA gene profiling, it cannot determine whether oral disease-associated taxa detected in stool represent viable bacteria, strain-level transfer from the oral cavity or stable engraftment in the distal gut. These signals should therefore be interpreted as an ecological footprint compatible with increased oral microbial input, transient passage or persistence and/or reduced colonisation resistance, rather than as direct evidence of long-term intestinal colonisation [8].

This interpretation is biologically plausible in light of previous work showing that partial gut oralisation can occur in clinical contexts that alter upper gastrointestinal or intestinal ecological barriers, including gastric acid suppression and proton-pump inhibitor exposure [21,22]. In selected settings, such as cirrhosis under long-term PPI therapy, gut oralisation has also been linked to markers of intestinal inflammation and increased gut permeability [23]. Thus, in PD, faecal enrichment of oral disease-linked taxa may reflect increased oral microbial input and/or a gut environment more permissive to their persistence, but cannot by itself distinguish greater oral exposure from altered gut colonisation resistance.

In PD, several clinical features may create conditions favouring increased oral microbial input to the gastrointestinal tract, including impaired oral clearance, xerostomia, dysphagia, gastroparesis and treatment-related effects that can worsen oral symptoms or oral-care routines [24,25]. Motor symptoms, notably tremor and bradykinesia, together with cognitive and affective symptoms, may further impair daily oral hygiene and limit access to regular dental care, thereby favouring the accumulation of caries- and periodontitis-associated oral communities. This interpretation is consistent with the increased burden of dental caries and oral disease reported in PD [1]. The high incidence of pneumonia in PD also illustrates the clinical relevance of impaired swallowing and altered handling of oral microbial contents, although this does not directly establish gut colonisation [26]. These relationships could not be robustly tested in the present re-analysis because most public 16S datasets lacked detailed dental, dietary, medication and clinical metadata and residual confounding by these factors cannot be excluded, highlighting the need for deeply phenotyped prospective cohorts.

A key observation was that oralisation-based stratification also revealed profile-related community patterns among controls. This suggests that gut oralisation is not unique to PD, but may instead reflect broader inter-individual variation in oral-gut microbial exchange, consistent with the strong influence of host-environment interactions on the gut microbiota [27]. This interpretation is also supported by evidence that oral-to-gut microbial transfer occurs in healthy individuals, varies substantially between subjects and supports the concept of the oral cavity as an endogenous reservoir for gut microbial strains [8]. In this view, PD may be associated with a greater frequency or extent of faecal oralisation in a subset of patients, shifting them further along an oralisation-related ecological gradient that is also detectable among controls, rather than inducing a distinct PD-specific gut microbiome state. The persistence of profile-related organisation after removal of the taxa used to define the original profiles, although attenuated, particularly in Bray–Curtis space, further suggests that these community differences were not entirely restricted to the taxa used for profile construction. The position of individuals along this shared oralisation-related gradient could plausibly be influenced by PD-related and treatment-associated changes in oral ecology, diet and feeding behaviour. In particular, increased preference for sweets or fast-acting carbohydrates, compulsive eating behaviours and weight gain reported in subsets of patients, including after subthalamic nucleus deep brain stimulation, may increase cariogenic pressure and favour caries-associated oral taxa [28–31]. However, because dietary intake, dental status and treatment history were not consistently available in public datasets, the contribution of these factors cannot be determined here and should be tested directly in prospective cohorts.

Among the taxa contributing to the cariogenic signal, *S. mutans* showed increased prevalence and abundance in PD samples. This observation is consistent with previous metagenomic evidence reporting increased faecal abundance of *S. mutans* in PD, including the large shotgun metagenomic study by Wallen et al., in which *S. mutans* was among the species most strongly enriched in PD [5]. The mechanistic plausibility of this association is further supported by recent experimental work showing that *S. mutans* can produce imidazole propionate through its urocanate reductase enzyme, UrdA and that this pathway can induce or exacerbate PD-relevant phenotypes in mouse models, including *α*-synuclein pathology, dopaminergic neuron loss and motor deficits [6]. These findings support further investigation of functional oral microbial pathways in PD, including UrdA-mediated imidazole propionate production and its clinical relevance in humans.

### eHOMD and SILVA played complementary roles in this analysis

eHOMD, a curated reference for the human aerodigestive/oral microbiome, was used to improve the detection and annotation of oral-associated taxa and to quantify oralisation signatures [12], whereas SILVA provided a broader, non-oral-specific taxonomic framework for community-level profiling [13]. This distinction is important because species-level taxonomic assignment remains inherently limited in 16S rRNA amplicon sequencing, particularly when data span different hypervariable regions. For this reason, species-level candidates are interpreted here as eHOMD-derived putative labels rather than definitive species identifications. Twelve eHOMD-derived putative labels had no corresponding assignment in SILVA, highlighting the expected differences in taxonomic resolution and database scope. Nevertheless, oralisation-related community patterns were also detectable in the SILVA-based analyses, indicating that the observed signal was not restricted to the eHOMD-specific taxonomic framework despite these database-dependent differences.

The enrichment pattern observed here was skewed toward caries/endodontic-associated taxa, including *S. mutans*, rather than toward a dominant periodontitis-associated signature. Consistent with this, the canonical periodontal pathogen *Porphyromonas gingivalis* was rarely detected in the present dataset across both taxonomic frameworks [32]. This does not exclude a contribution of periodontal dysbiosis to oral–gut microbial exchange, but suggests that the faecal oralisation signal captured here was not primarily driven by classical periopathogens. This interpretation is also consistent with some experimental periodontitis models, in which periodontal infection induced marked periodontal microbiota dysbiosis and systemic immune-metabolic alterations without major effects on gut microbiota composition [33]. One possible explanation is that oral taxa differ in their ability to tolerate the physicochemical barriers encountered during upper gastrointestinal transit [8]. In PD, delayed gastric emptying and other upper gastrointestinal disturbances could further modify this selective bottleneck [24]. Cariogenic bacteria such as *S. mutans* are acidogenic and aciduric, traits that could plausibly favour persistence during passage through acidic or fluctuating pH environments [34,35]. By contrast, the limited detection of classical periodontal pathogens in this dataset may reflect differences in oral abundance and shedding, niche specificity, survival during gastrointestinal transit or the taxonomic resolution of 16S rRNA sequencing.

### Strengths, limitations and implications

The main strengths of this study include the scale of the pooled analysis, harmonised reprocessing of raw sequencing data and accounting for key study-level and technical sources of heterogeneity, including BioProject structure and 16S rRNA gene region where appropriate. In this context, oral-enrichment profiles provided an exploratory framework for identifying subgroup-related patterns within the PD gut microbiome that were less apparent from global PD-control comparisons alone. The associated beta-diversity effects were modest in magnitude and are therefore interpreted as components of broader microbiome heterogeneity rather than dominant drivers of community structure. Limitations are primarily related to the nature of public multi-cohort re-analyses. Some otherwise relevant studies could not be included because raw reads or valid repository identifiers were unavailable. Also, harmonised metadata on age, sex, BMI, disease duration, oral health, swallowing function, constipation and other gastrointestinal symptoms, medication exposure, diet and disease severity were often lacking across studies. Geographic location was closely linked to BioProject, since individual cohorts were generally recruited within a specific country. Its independent effect could therefore not be reliably separated from other BioProject-level differences, similarly to the 16S rRNA gene region. These limitations restricted direct testing of the mechanisms that may underlie gut oralisation and residual confounding cannot be excluded. Control-group definitions and matching procedures also varied across studies, reflecting the design of the original cohorts and commonly including household, spouse/partner and community controls in PD analyses. These differences are inherent to public multi-cohort re-analyses and may contribute residual heterogeneity. Although BioProject structure was accounted for in the pooled analyses, this cannot substitute for adjustment of unavailable individual-level covariates [36–39].

Oral-enrichment profiles were defined using pragmatic, control-derived thresholds and should therefore be interpreted as exploratory ecological strata rather than diagnostic categories. Nevertheless, the consistency of the patterns observed with MaAsLin2 and ANCOM-BC2, together with the SILVA-based community analyses, indicates that the oralisation-related pattern was not restricted to a single statistical approach or taxonomic framework. Importantly, the external literature-derived panel analysis provided independent support for the caries/endodontic-associated signal, while removal of the taxa used to construct the original profiles showed that some profile-related community organisation persisted beyond the defining taxa. Overall, oral-disease annotations provided a biologically interpretable framework for exploring gut microbiome variation. SILVA-based ordinations further indicated that this variation was better described as a compositional gradient than as discrete, fully separated clusters. Together, these findings suggest that gut microbiota variation in PD is partly structured along an oralisation-related axis, with a prominent caries/endodontic-associated component. This pattern may represent a potential indirect marker of altered oral ecosystem status in a subset of PD patients [1,2,40], rather than evidence of a direct causal driver or a distinct PD-specific microbiome state.

## Conclusion

In conclusion, this pooled re-analysis supports the presence of a reproducible gut oralisation-associated signal in a substantial subset of patients with PD, with caries/endodontic-associated taxa contributing prominently to the PD-associated signal. Rather than defining a single PD-specific microbiome state, this pattern appeared to reflect an oralisation-related gradient within the broader gut microbiome. These findings are consistent with reports of poorer oral health in PD and suggest that variation in the faecal representation of oral-associated taxa may represent one component of gut microbiome heterogeneity and a potential indirect marker of altered oral ecosystem status. Although several cariogenic taxa, including *S. mutans*, showed increased prevalence or abundance in PD, the oralisation signal was not restricted to a single organism. Instead, it involved multiple oral-associated taxa and was concentrated in particular patient subgroups. Longitudinal and interventional studies integrating standardised dental and periodontal phenotyping, oral microbiome assessment, dietary information, detailed neurological metadata and strain-resolved microbiome profiling will be required to clarify the origin of the faecal oral-associated signal and determine its relationship with individual oral health and other clinical and lifestyle factors, as well as its clinical relevance. Overall, these results suggest that oral health, although often overlooked, should be considered more systematically in PD microbiome research and clinical follow-up.

## Supplementary Material

Supplementary_Material new.docx Supplementary_Material new.docx

Supplemental Material Fig_S6_23_Sep_2026_15_07_AU.pdf

Supplemental Material Fig_S5_23_Sep_2026_15_04_AU.pdf

Supplemental Material Fig_S4_23_Sep_2026_14_08_AU.pdf

Supplemental Material Fig_S3_23_Sep_2026_14_30_AU.pdf

Supplemental Material Fig_S2_23_Sep_2026_13_41_AU.pdf

Supplemental Material Fig_S1_23_Sep_2026_13_37_AU.pdf

Supplementary Material Table_S8A_S8B

## AI

The authors report generative AI was not used in their research or preparation of this manuscript.
