# Supplementary material for: Gut oralisation signatures in Parkinson's disease: an integrative multi-study analysis
Source: J Oral Microbiol. 2026 Oct 11;18(1):2737700. doi: 10.1080/20002297.2026.2737700 (PMC13656644; doi:10.1080/20002297.2026.2737700)
Supplement: Supplementary file 1 [file ZJOM_A_2737700_SM8876.pdf]

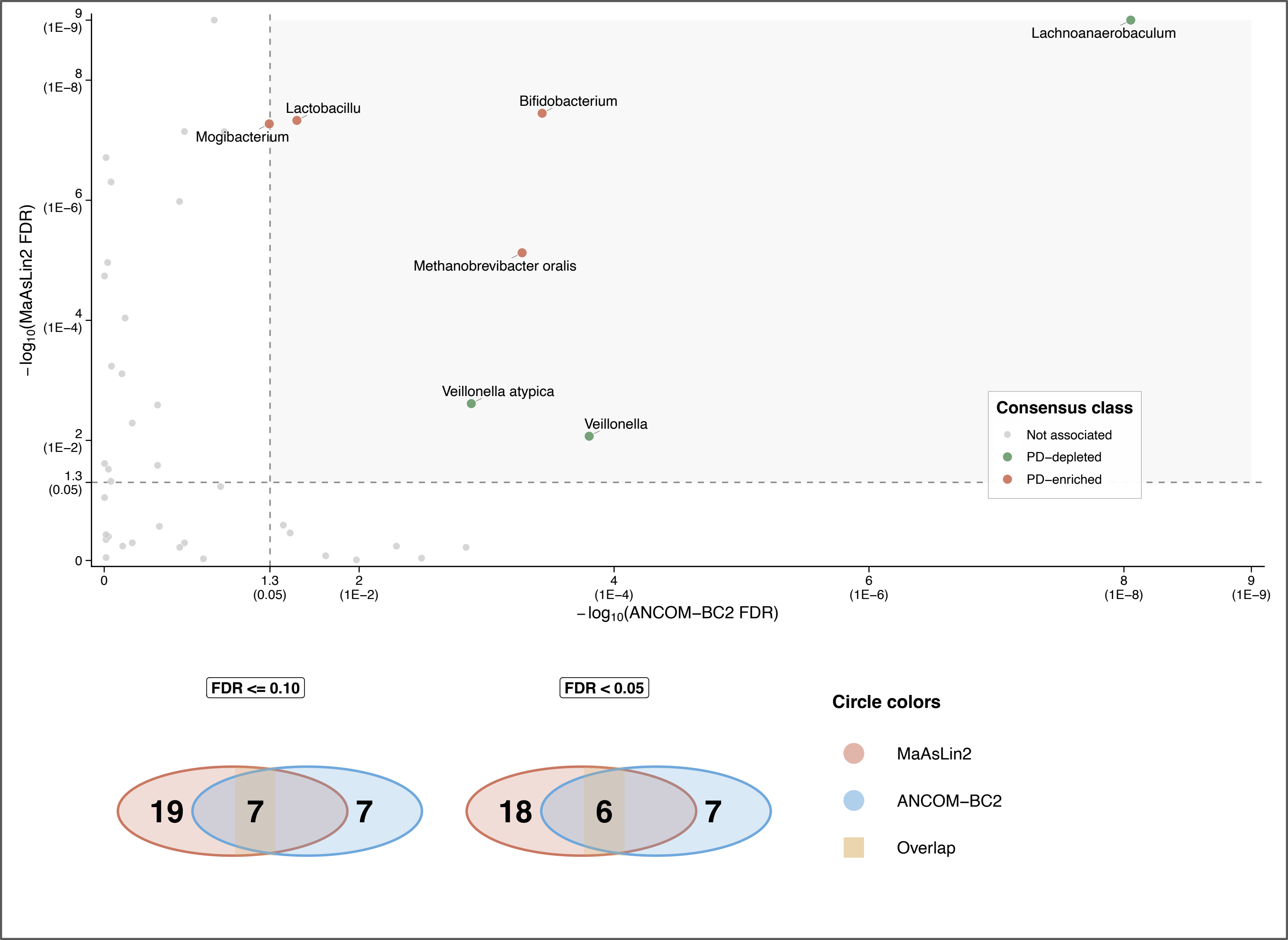

Lachnoanaerobaculum
Bifidobacterium
Lactobacillu
Mogibacterium
Methanobrevibacter oralis
Veillonella atypica
Veillonella
Consensus class
Not associated
PD−depleted
PD−enriched
−log10(MaAsLin2 FDR)
−log10(ANCOM−BC2 FDR)
9 (1E−9)
8 (1E−8)
6 (1E−6)
4 (1E−4)
2 (1E−2)
1.3 (0.05)
0
FDR <= 0.10
FDR < 0.05
19
7
7
18
6
7
Circle colors
MaAsLin2
ANCOM−BC2
Overlap
