# Supplementary material for: Gut oralisation signatures in Parkinson's disease: an integrative multi-study analysis
Source: J Oral Microbiol. 2026 Oct 11;18(1):2737700. doi: 10.1080/20002297.2026.2737700 (PMC13656644; doi:10.1080/20002297.2026.2737700)
Supplement: Supplementary file 2 [file ZJOM_A_2737700_SM8877.pdf]

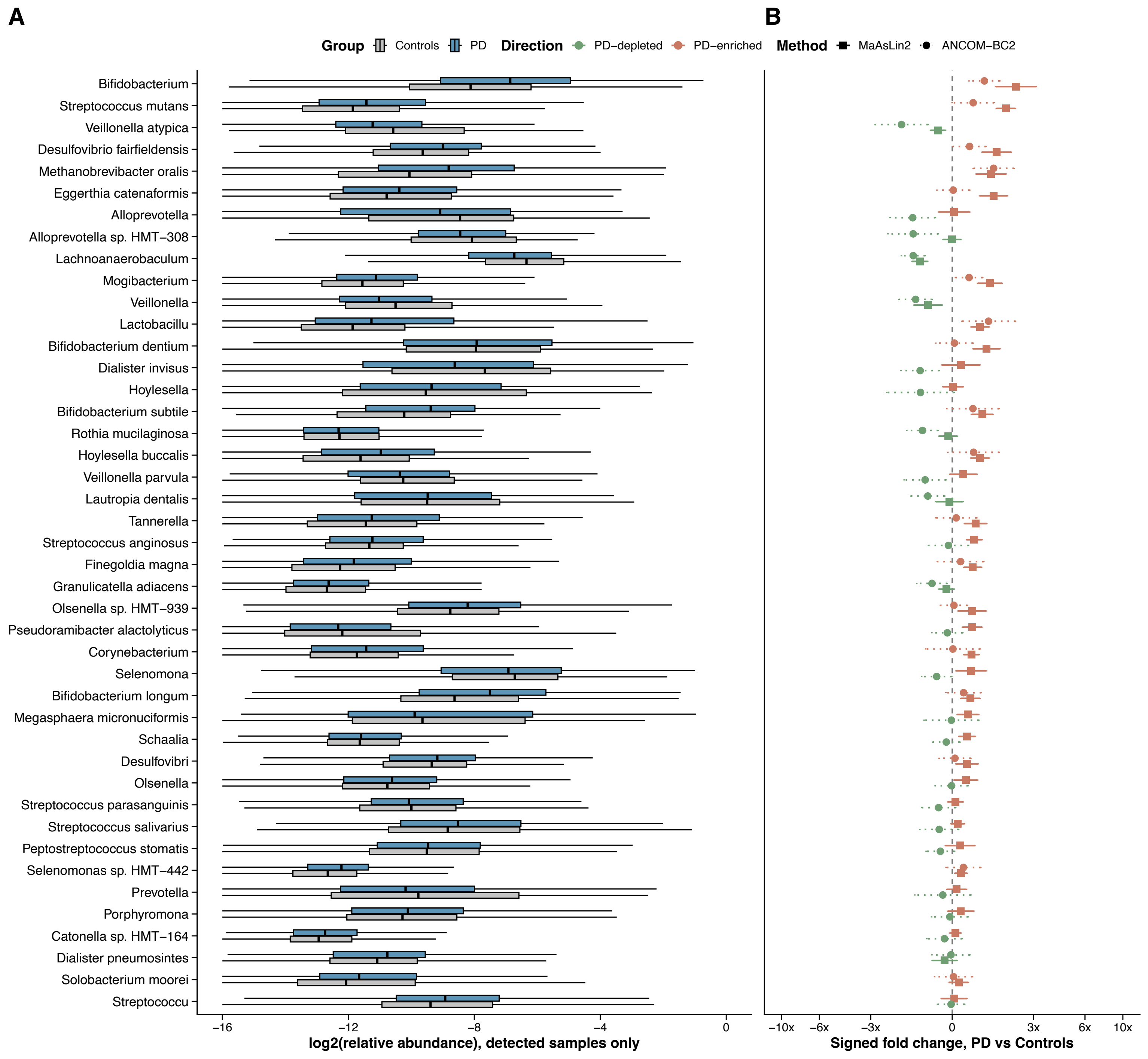
A
B
Group
Controls
PD
Direction
PD−depleted
PD−enriched
Method
MaAsLin2
ANCOM−BC2
Bifidobacterium
Streptococcus mutans
Veillonella atypica
Desulfovibrio fairfieldensis
Methanobrevibacter oralis
Eggerthia catenaformis
Alloprevotella
Alloprevotella sp. HMT−308
Lachnoanaerobaculum
Mogibacterium
Veillonella
Lactobacillu
Bifidobacterium dentium
Dialister invisus
Hoylesella
Bifidobacterium subtile
Rothia mucilaginosa
Hoylesella buccalis
Veillonella parvula
Lautropia dentalis
Tannerella
Streptococcus anginosus
Finegoldia magna
Granulicatella adiacens
Olsenella sp. HMT−939
Pseudoramibacter alactolyticus
Corynebacterium
Selenomona
Bifidobacterium longum
Megasphaera micronuciformis
Schaalia
Desulfovibri
Olsenella
Streptococcus parasanguinis
Streptococcus salivarius
Peptostreptococcus stomatis
Selenomonas sp. HMT−442
Prevotella
Porphyromona
Catonella sp. HMT−164
Dialister pneumosintes
Solobacterium moorei
Streptococcu
−16
−12
−8
−4
0
log2(relative abundance), detected samples only
−10x
−6x
−3x
0
3x
6x
10x
Signed fold change, PD vs Controls
