# Supplementary material for: Gut oralisation signatures in Parkinson's disease: an integrative multi-study analysis
Source: J Oral Microbiol. 2026 Oct 11;18(1):2737700. doi: 10.1080/20002297.2026.2737700 (PMC13656644; doi:10.1080/20002297.2026.2737700)
Supplement: Supplementary file 3 [file ZJOM_A_2737700_SM8880.pdf]

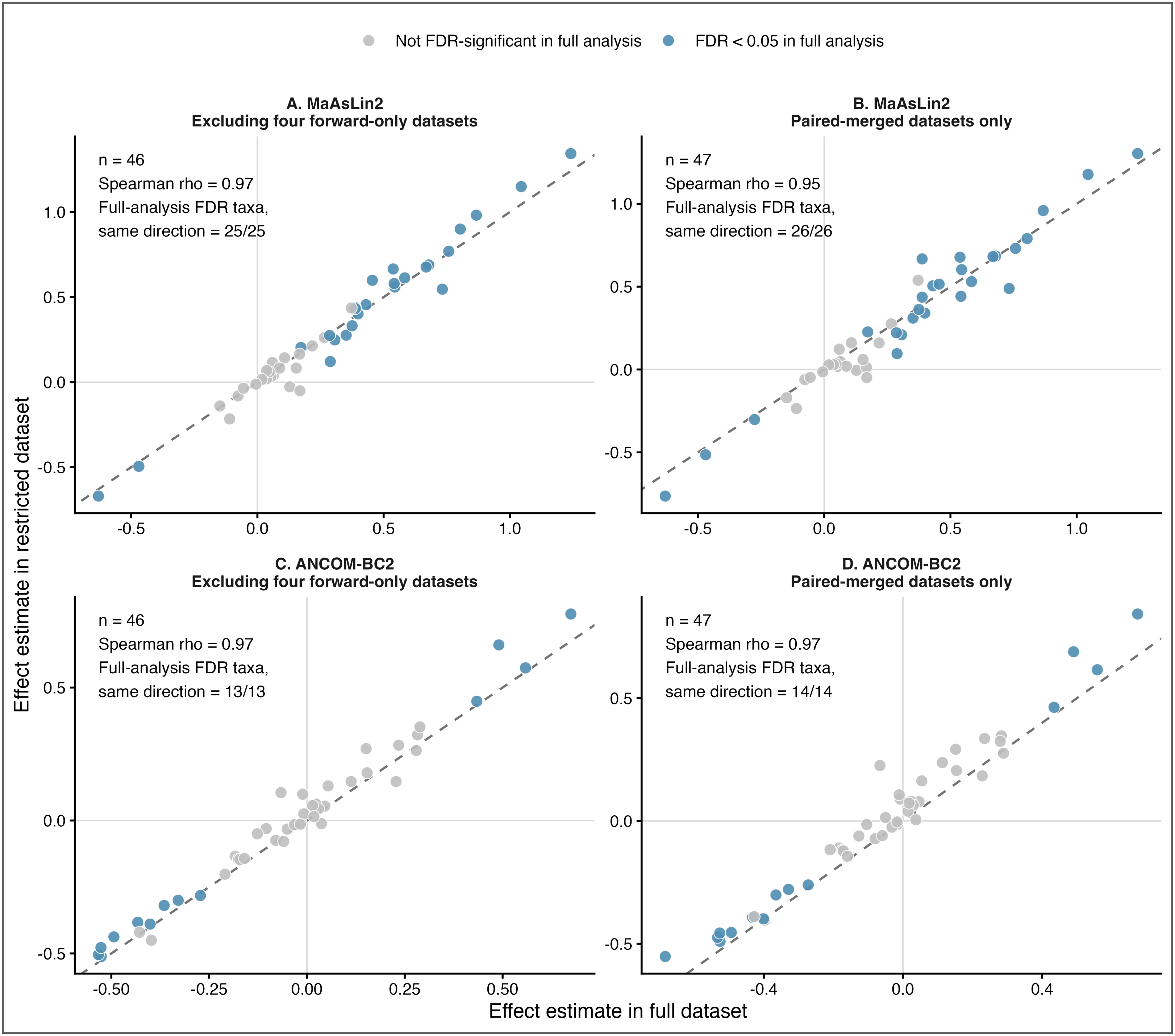
Not FDR-significant in full analysis
FDR < 0.05 in full analysis
A. MaAsLin2
Excluding four forward-only datasets
n = 46
Spearman rho = 0.97
Full-analysis FDR taxa,
same direction = 25/25
B. MaAsLin2
Paired-merged datasets only
n = 47
Spearman rho = 0.95
Full-analysis FDR taxa,
same direction = 26/26
C. ANCOM-BC2
Excluding four forward-only datasets
n = 46
Spearman rho = 0.97
Full-analysis FDR taxa,
same direction = 13/13
D. ANCOM-BC2
Paired-merged datasets only
n = 47
Spearman rho = 0.97
Full-analysis FDR taxa,
same direction = 14/14
Effect estimate in restricted dataset
Effect estimate in full dataset
