# Supplementary material for: Gut oralisation signatures in Parkinson's disease: an integrative multi-study analysis
Source: J Oral Microbiol. 2026 Oct 11;18(1):2737700. doi: 10.1080/20002297.2026.2737700 (PMC13656644; doi:10.1080/20002297.2026.2737700)
Supplement: Supplementary file 4 [file ZJOM_A_2737700_SM8881.pdf]

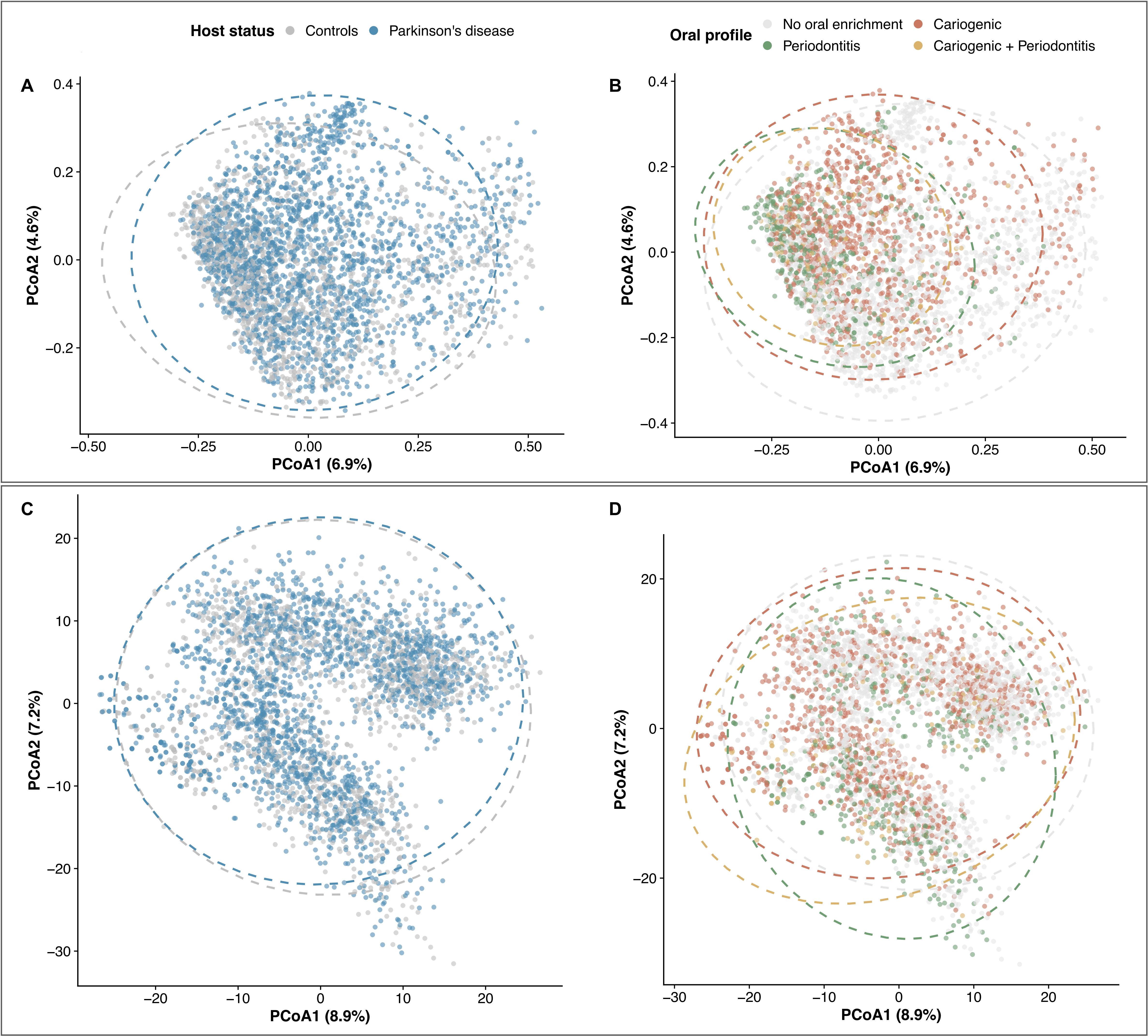
Host status
Controls
Parkinson's disease
Oral profile
No oral enrichment
Cariogenic
Periodontitis
Cariogenic + Periodontitis
A
B
C
D
PCoA1 (6.9%)
PCoA2 (4.6%)
PCoA1 (8.9%)
PCoA2 (7.2%)
