# Supplementary material for: Gut oralisation signatures in Parkinson's disease: an integrative multi-study analysis
Source: J Oral Microbiol. 2026 Oct 11;18(1):2737700. doi: 10.1080/20002297.2026.2737700 (PMC13656644; doi:10.1080/20002297.2026.2737700)
Supplement: Supplementary file 5 [file ZJOM_A_2737700_SM8882.pdf]

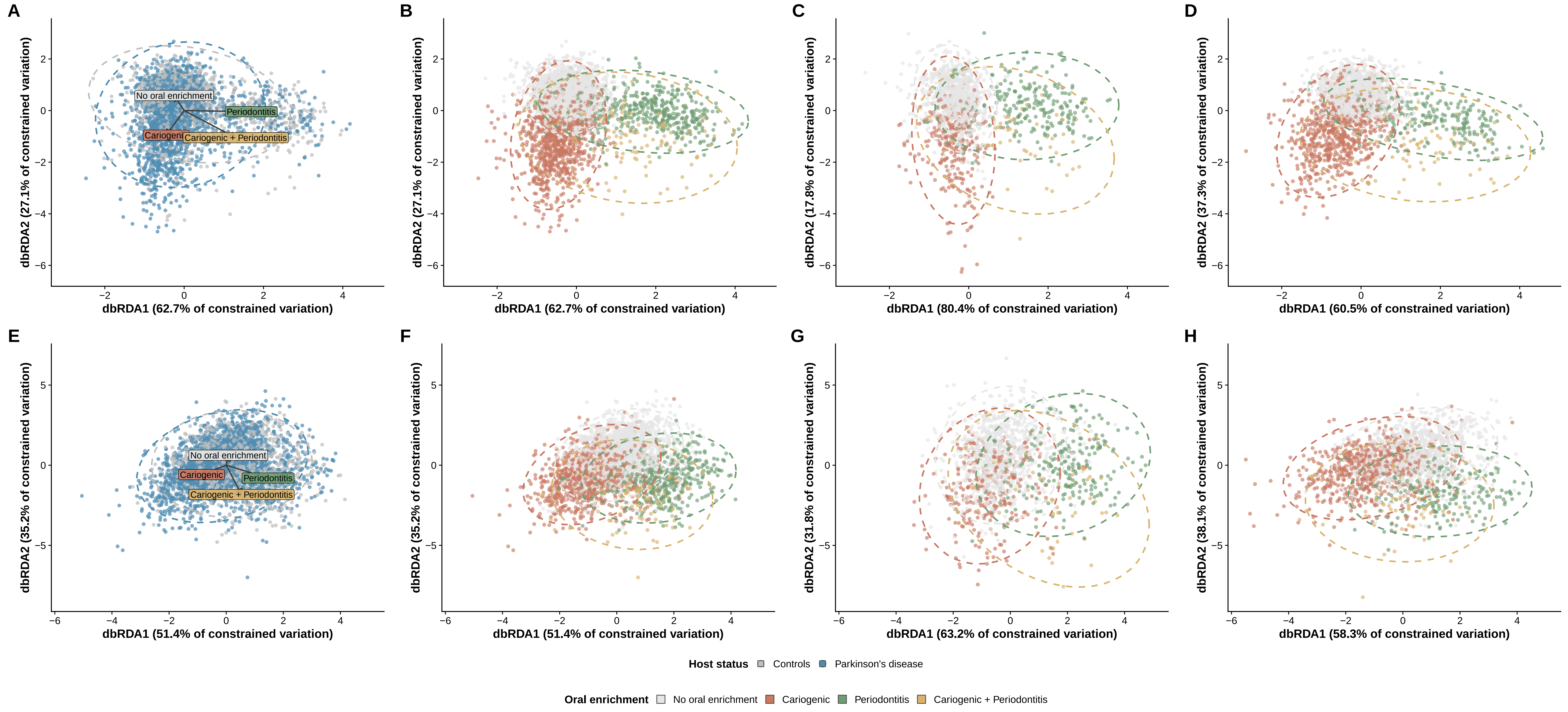
A
B
C
D
E
F
G
H
No oral enrichment
Periodontitis
Cariogenic
Cariogenic + Periodontitis
dbRDA1 (62.7% of constrained variation)
dbRDA2 (27.1% of constrained variation)
dbRDA1 (80.4% of constrained variation)
dbRDA2 (17.8% of constrained variation)
dbRDA1 (60.5% of constrained variation)
dbRDA2 (37.3% of constrained variation)
dbRDA1 (51.4% of constrained variation)
dbRDA2 (35.2% of constrained variation)
dbRDA1 (63.2% of constrained variation)
dbRDA2 (31.8% of constrained variation)
dbRDA1 (58.3% of constrained variation)
dbRDA2 (38.1% of constrained variation)
Host status
Controls
Parkinson's disease
Oral enrichment
No oral enrichment
Cariogenic
Periodontitis
Cariogenic + Periodontitis
