# Supplementary material for: Gut oralisation signatures in Parkinson's disease: an integrative multi-study analysis
Source: J Oral Microbiol. 2026 Oct 11;18(1):2737700. doi: 10.1080/20002297.2026.2737700 (PMC13656644; doi:10.1080/20002297.2026.2737700)
Supplement: Supplementary file 6 [file ZJOM_A_2737700_SM8883.pdf]

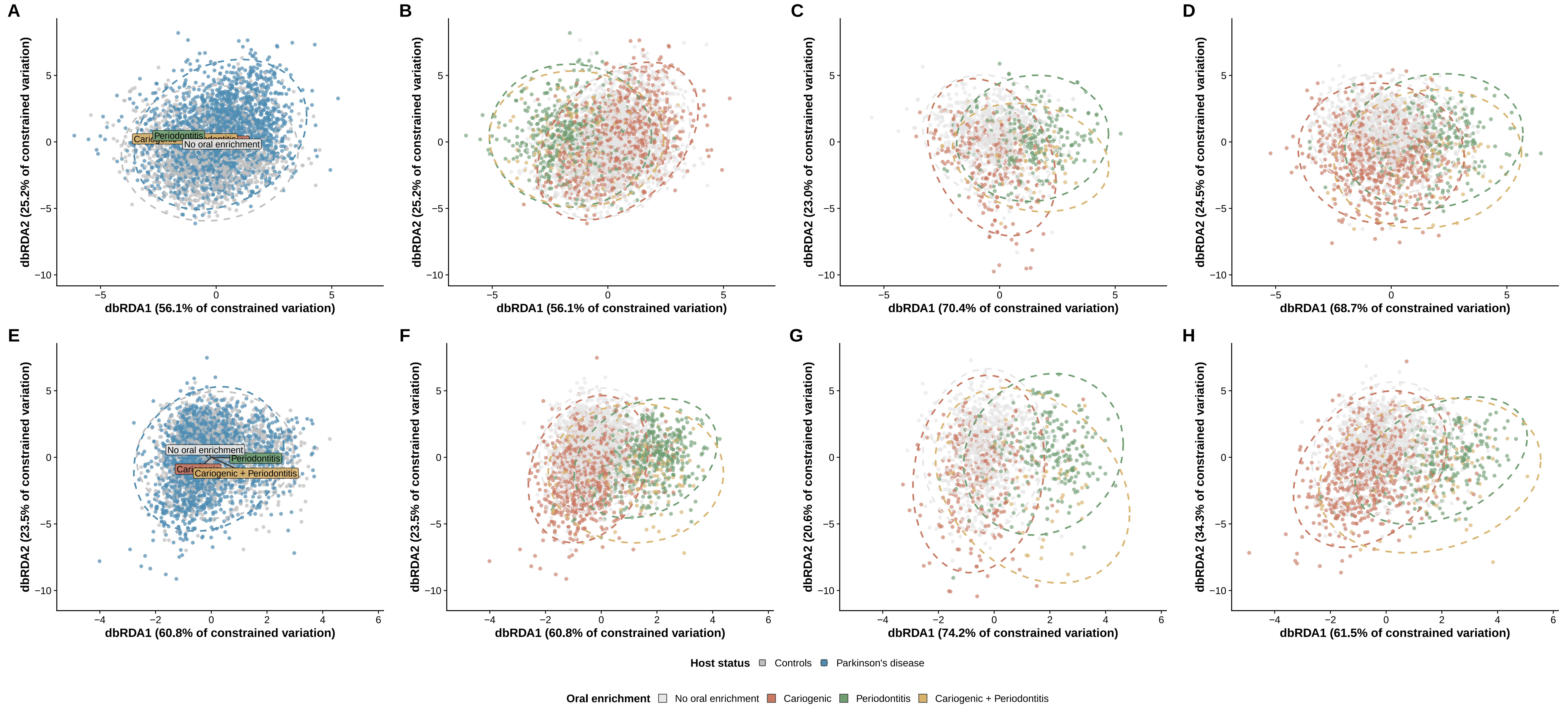

A
Periodontitis
No oral enrichment
dbRDA2 (25.2% of constrained variation)
dbRDA1 (56.1% of constrained variation)
B
dbRDA2 (25.2% of constrained variation)
dbRDA1 (56.1% of constrained variation)
C
dbRDA2 (23.0% of constrained variation)
dbRDA1 (70.4% of constrained variation)
D
dbRDA2 (24.5% of constrained variation)
dbRDA1 (68.7% of constrained variation)
E
No oral enrichment
Periodontitis
Cariogenic + Periodontitis
dbRDA2 (23.5% of constrained variation)
dbRDA1 (60.8% of constrained variation)
F
dbRDA2 (23.5% of constrained variation)
dbRDA1 (60.8% of constrained variation)
G
dbRDA2 (20.6% of constrained variation)
dbRDA1 (74.2% of constrained variation)
H
dbRDA2 (34.3% of constrained variation)
dbRDA1 (61.5% of constrained variation)
Host status
Controls
Parkinson's disease
Oral enrichment
No oral enrichment
Cariogenic
Periodontitis
Cariogenic + Periodontitis
