# Supplementary material for: Gut oralisation signatures in Parkinson's disease: an integrative multi-study analysis
Source: J Oral Microbiol. 2026 Oct 11;18(1):2737700. doi: 10.1080/20002297.2026.2737700 (PMC13656644; doi:10.1080/20002297.2026.2737700)
Supplement: Supplementary file 7 [file ZJOM_A_2737700_SM4929.docx]

**SUPPLEMENTARY DATA**

**Supplementary Methods**

**Study identification, data retrieval and preprocessing.** PubMed was searched without year restriction, with the last search performed on 30 September 2025, using the following query: “parkinson” AND “gut” AND “microbiota” NOT “review” AND english[Filter] NOT “mouse” NOT “meta-analysis” NOT “systematic reviews”. Screening and eligibility assessment were performed using predefined inclusion and exclusion criteria, following PRISMA 2020 recommendations where applicable. Studies were excluded at the full-text stage when they used non-16S rRNA gene sequencing designs, including shotgun-only metagenomics, included non-stool samples, lacked unambiguous Parkinson’s disease (PD) and control group annotations, had restricted access to raw sequencing reads, or provided invalid or unresolved repository identifiers. For all included studies, accession numbers from BioProject, SRA or ENA repositories and available sample-level metadata were compiled into a study manifest (Table S1).

Raw sequencing reads were retrieved from SRA or ENA using either Galaxy Europe, through the “Download and Extract Reads in FASTQ format from NCBI SRA” tool, or the SRA Toolkit using prefetch and fasterq-dump. Each dataset was processed independently in QIIME 2 (v2025.10). Primer and adapter sequences were removed using Cutadapt with dataset-specific primer information (Table S2). Denoising was performed with DADA2, using paired-end or single-end processing depending on sequencing design and read-quality profiles. Truncation parameters were defined separately for each dataset to retain high-quality sequence regions while preserving sufficient overlap for paired-end merging when applicable (Table S2). Four datasets originally generated as paired-end (PRJNA822998, PRJNA822715, PRJNA975118 and PRJEB36138) required forward-only processing. In these datasets, reverse-read quality declined substantially toward the 3′ end. Retaining longer reverse reads would have required keeping low-quality bases, whereas more stringent truncation reduced the forward–reverse overlap required for reliable merging and/or resulted in substantial read loss. To maintain consistent quality criteria across studies, these datasets were therefore denoised using forward reads only rather than relaxing DADA2 quality filtering criteria. This should be distinguished from PRJEB14674, which was generated as single-end in the original study. Samples with fewer than 5,000 reads after denoising were excluded from downstream analyses. Feature tables and representative sequences were generated independently for each dataset before downstream pooling. Sample metadata were harmonized across datasets to define sample identifier, case-control status (PD vs Controls), BioProject ID, dataset ID and amplified 16S rRNA gene region. Only samples annotated as PD or controls and passing quality-control filters were retained for pooled analyses.

**Taxonomic assignment with eHOMD and SILVA.** Taxonomic assignment was performed using both eHOMD (v16.01; oral-curated) and SILVA (SSURef NR99, release 138.1, 99% identity) reference databases. eHOMD was used to improve the resolution of oral-associated taxa and to support oral disease–linked taxon annotation, whereas SILVA was used for broader gut community analyses, including alpha-diversity, beta-diversity and constrained ordination. Region-specific naïve Bayes classifiers were trained after in silico extraction of reference sequences matching the amplified 16S rRNA gene region of each dataset. Taxonomy was assigned independently for each dataset using the corresponding region-specific classifier before generating taxonomic tables aggregated at the lowest resolved taxonomic level. Taxonomic tables were exported and harmonized across datasets. For pooled analyses, taxonomic features were matched across datasets using standardized feature names and sample-level metadata. Separate pooled tables were generated for eHOMD-based analyses of oral-associated taxa and SILVA-based analyses of overall community diversity.

**Differential abundance analyses.** Statistical analyses were performed in R version 4.5.1. Differential abundance was assessed using two complementary frameworks, MaAsLin2 and ANCOM-BC2. For both approaches, case-control status was modelled as the primary variable of interest, amplified 16S rRNA gene region was included as a fixed effect, and BioProject ID was included as a random intercept to account for between-project heterogeneity. Controls were used as the reference category for case-control status, and V3–V4 was used as the reference level for the 16S rRNA gene region.

For MaAsLin2, the pooled eHOMD taxonomic count table was used as input. Metadata and feature tables were aligned by sample identifier, retaining samples annotated as controls or PD and with region and project information. Taxa were filtered before modelling by retaining only features detected in at least 10% of samples. MaAsLin2 was run using total-sum scaling normalization (normalization = "TSS"), log transformation (transform = "LOG") and linear modelling (analysis_method = "LM"). Effect estimates were extracted for the PD versus Controls contrast. P-values were adjusted using the Benjamini–Hochberg (BH) procedure, and q < 0.05 was considered statistically significant.

ANCOM-BC2 was applied to the corresponding pooled eHOMD taxonomic count table as a compositional bias-corrected complementary analysis. Metadata and count matrices were strictly aligned by sample identifier, retaining only samples annotated as controls or PD and with 16S rRNA gene region and BioProject ID. The ANCOM-BC2 model included host_status (case-control status; PD vs Controls) and region_16S as fixed effects and (1 | project_id) as the random-effect structure. Taxa were filtered using a 10% prevalence threshold (prv_cut = 0.10), with no additional library-size filtering (lib_cut = 0). Pseudo-count sensitivity analysis was enabled, negative lower-bound correction was applied, and multiple testing was controlled using BH correction, with q < 0.05 considered statistically significant.

**Sensitivity analysis for read-processing strategy.** Because this study is a harmonized reanalysis of publicly available datasets, sequencing design and raw-read quality were inherited from the original studies. To assess whether datasets requiring forward-only processing influenced the pooled PD-versus-control differential-abundance results, MaAsLin2 and ANCOM-BC2 were rerun after excluding the four originally paired-end datasets that required forward-only processing (PRJNA822998, PRJNA822715, PRJNA975118, and PRJEB36138). This sensitivity dataset comprised 3,231 samples (1,930 PD and 1,301 controls). As a secondary, more restrictive analysis, the models were repeated using only datasets successfully processed as paired-end, additionally excluding PRJEB14674, which was originally generated as single-end. This subset comprised 2,901 samples (1,730 PD and 1,171 controls). Effect estimates for PD versus controls obtained in each sensitivity analysis were compared with those from the primary pooled analysis using Spearman rank correlations across taxa available in both models. For taxa significant in the primary analysis and retained in the corresponding sensitivity model, concordance in effect direction and retention of FDR < 0.05 were also assessed. Because processing mode was confounded with dataset/BioProject identity, differences between forward-only and paired-merged datasets were not interpreted as effects attributable to processing mode.

**Annotation of oral disease–associated taxa.** For the original exploratory oral-enrichment framework, differential abundance outputs from MaAsLin2 and ANCOM-BC2 were annotated using oral disease–associated categories based on eHOMD annotations and supporting literature. Taxa were classified into two mutually exclusive categories: caries/endodontic-associated taxa and periodontitis/subgingival plaque-associated taxa. All taxa identified in the differential abundance outputs were manually reviewed against eHOMD annotations and the literature and assigned to curated annotation ecological/clinical categories, including caries/endodontic-associated taxa, periodontitis/subgingival plaque-associated taxa, other oral/dental taxa, and gut-associated or non-oral taxa. Only taxa classified as caries/endodontic-associated or periodontitis/subgingival plaque-associated were used for disease-specific score construction; the remaining taxa were retained in the annotated output tables but were not included in the enrichment scores. Annotated result tables were then used for downstream enrichment-score calculation, oral-profile classification and supplementary table generation.

**Assessment of concordance between MaAsLin2 and ANCOM-BC2.** To evaluate the concordance of PD-associated oral taxa across differential-abundance methods, MaAsLin2 and ANCOM-BC2 outputs were merged by taxon after normalization of taxonomic identifiers. For each method, case-control status contrast corresponding to PD versus Controls was extracted, and effect directions were compared to identify concordant enrichment or depletion in PD. Taxa were considered consensus-associated when they reached FDR < 0.05 in one method and FDR ≤ 0.10 in the other method, with concordant direction of effect. Taxa with positive effect estimates in both methods were classified as PD-enriched, whereas taxa with negative effect estimates in both methods were classified as PD-depleted. Method concordance was visualized by plotting −log10 FDR values from MaAsLin2 and ANCOM-BC2 and by summarizing method overlap using pseudo- Venn-style diagrams at FDR ≤ 0.10 and FDR < 0.05.

**Visualization of differential-abundance results and method concordance.** Relative abundances of oral disease–linked taxa were plotted after log2 transformation, using only samples in which the corresponding taxon was detected. Model-derived signed fold changes were calculated from MaAsLin2 and ANCOM-BC2 effect estimates, with positive values indicating enrichment in PD and negative values indicating depletion in PD. Horizontal intervals were derived from model standard errors, and fold changes were capped at ±10-fold for visualization. Taxa were ordered according to the largest absolute effect observed across the two methods.

**Compilation of oral disease–associated taxa.** To generate Table 3, annotated MaAsLin2 and ANCOM-BC2 outputs were merged by taxon after normalization of taxonomic identifiers. Only taxa present in both differential abundance outputs and significantly associated with case-control status in at least one method using the prespecified threshold of FDR < 0.05 were retained. Oral disease annotations were consolidated across methods and grouped into caries/endodontic-associated or periodontitis/subgingival plaque-associated categories. Retained taxa were matched back to the pooled eHOMD abundance table to calculate descriptive abundance summaries. For each taxon, prevalence, mean relative abundance and standard deviation were calculated separately in PD and control samples. The final table reports the taxon name, oral disease category, MaAsLin2 effect estimate and FDR, ANCOM-BC2 log-fold-change estimate and FDR, method-specific significance status, group-specific prevalence and abundance summaries, and references supporting the oral disease association. MaAsLin2 coefficients correspond to the PD versus Controls contrast. ANCOM-BC2 estimates correspond to the case-control status log-fold-change estimate for PD relative to Controls. Because ANCOM-BC2 reports log-fold-change estimates rather than raw fold changes, these values were reported as effect estimates rather than untransformed fold changes.

**Oral-enrichment profile construction.** For the original exploratory oral-enrichment framework, separate enrichment scores were calculated using MaAsLin2- and ANCOM-BC2-derived taxa sets. For each method, taxa annotated as caries/endodontic-associated or periodontitis/subgingival plaque-associated and associated with case-control status at a prespecified threshold of q < 0.05 were retained for score construction. Retained taxa were then matched to the pooled eHOMD relative-abundance table. For each sample, a caries/endodontic enrichment score and a periodontitis enrichment score were calculated as the cumulative relative abundance of the retained taxa within each category. Method-specific thresholds were defined as the mean score observed among controls for the corresponding category. Samples were classified into one of four mutually exclusive profiles according to whether their caries/endodontic and/or periodontitis score exceeded the corresponding control-derived threshold: no oral enrichment, caries/endodontic enrichment, periodontitis enrichment, or combined caries/endodontic and periodontitis enrichment. Profile distributions were summarized separately for MaAsLin2- and ANCOM-BC2-derived taxa sets. Differences in the distribution of oral-enrichment profiles between controls and PD samples were assessed using 2 × 4 contingency tables. Pearson’s χ² tests were used for global comparisons because all expected cell counts exceeded five. Post-hoc profile-wise comparisons were performed by comparing each enrichment profile against all remaining profiles using Fisher’s exact tests. P-values were adjusted using the BH procedure, and Cramér’s V was reported as a standardized effect size for the global contingency-table analyses.

**Cariogenic-to-periodontitis oral balance.** As a complementary continuous analysis within the original exploratory oral-enrichment framework, a cariogenic-to-periodontitis oral balance was calculated for each sample using the MaAsLin2- and ANCOM-BC2-derived taxa sets. Retained taxa were grouped according to their oral disease annotation. The balance was defined as the difference between the mean log-transformed relative abundance of caries/endodontic-associated taxa and the mean log-transformed relative abundance of periodontitis-associated taxa, after addition of a 1 × 10⁻⁶ pseudo-count. The standard ilr balance scaling factor, √(rs/(r+s)), was applied, where r and s correspond to the number of retained caries/endodontic-associated and periodontitis-associated taxa, respectively. Positive values indicate a shift toward greater relative representation of caries/endodontic-associated taxa, whereas negative values indicate a shift toward greater relative representation of periodontitis-associated taxa. Balance distributions were compared between controls and PD samples using Wilcoxon rank-sum tests, followed by BH correction across methods. Distributions were visualized using violin plots overlaid with boxplots and individual sample points.

**Case-control status versus amplicon-region sensitivity analysis.** To assess whether oral disease–linked associations were potentially influenced by 16S rRNA gene amplicon-region effects, a heuristic association-pattern analysis was performed using MaAsLin2 outputs. For each taxon, the MaAsLin2 coefficient and FDR associated with case-control status were extracted, with positive coefficients corresponding to higher abundance in PD relative to controls. Amplicon-region effects were assessed from MaAsLin2 coefficients associated with region_16S. For each taxon, the region contrast with the lowest FDR was selected, and the reported region effect size, direction and FDR were taken from that same contrast. Using FDR < 0.05 as the significance threshold, taxa significant for case-control status but not for region were classified as PD-associated; taxa significant for region but not case-control status were classified as region-associated; and taxa significant for both were classified as mixed PD/region-associated.

**PD-independent literature-derived oral disease panels.** To address potential circularity in the original oral-enrichment profiles, we performed a sensitivity analysis using oral disease-associated taxa selected exclusively from external literature, independently of their association with PD in the present dataset. Two mutually exclusive panels were defined a priori: a caries/endodontic panel (8 taxa) and a periodontitis panel (25 taxa). After panel definition, taxa were checked for representation in the pooled eHOMD dataset. All 8 caries/endodontic taxa and 18 of the 25 periodontitis taxa were represented and available for quantitative analysis. Taxonomic mappings were explicitly checked, and absent taxa were not included in the quantitative analysis. For each sample, reads assigned to panel taxa were summed and compared with reads assigned to the remaining eHOMD community using beta-binomial mixed-effects models (glmmTMB): cbind(panel_count, other_count) ~ host_status + region_16S + (1 | project_id). PD-*versus*-Control effects were expressed as odds ratios obtained by exponentiating the host_status coefficient. An OR > 1 indicates that, relative to the remainder of the eHOMD community, the odds for a read to belong to the specified panel were higher in PD than in Controls; an OR < 1 indicates the opposite. These estimates therefore reflect relative panel representation in PD *versus* Controls, accounting for 16S rRNA gene region as a fixed effect and BioProject as a random effect, rather than absolute bacterial abundance.

Sensitivity analyses were performed to assess the robustness of the literature-derived panel associations. First, models were repeated after restriction to BioProjects containing both PD and Control samples, thereby excluding the PD-only BioProjects. Second, analyses were repeated separately within the two predominant 16S rRNA gene regions, V3–V4 and V4, to evaluate whether the observed associations were reproducible across the main amplicon regions represented in the dataset. Finally, leave-one-taxon-out analyses were performed by removing each represented taxon in turn and refitting the model, to determine whether the panel-level association depended disproportionately on a single species. Panel composition was kept fixed throughout and was not modified on the basis of these sensitivity results.

**Alpha-diversity analysis.** Alpha-diversity analyses were performed using SILVA-derived taxonomic profiles aggregated at the lowest resolved taxonomic level and calculated in R using the ‘vegan’ package (1). Observed richness, Shannon diversity and Pielou’s evenness were calculated for each sample. These metrics were compared between PD and Control samples globally and after stratification by the exploratory oral-enrichment profiles. Group comparisons were performed using Wilcoxon rank-sum tests. P-values were adjusted using the BH procedure to account for multiple testing across alpha-diversity metrics and profile strata. These analyses were used to determine whether case-control status or oral-enrichment profiles were associated with broad changes in taxonomic richness, overall diversity or community evenness.

**Beta-diversity analysis.** Bray–Curtis beta-diversity analyses were performed using SILVA-derived relative-abundance taxonomic profiles. The distances were calculated using vegan::vegdist (2). A principal coordinates analysis (PCoA) was performed to visualize overall community variation according to case-control status, with samples coloured as controls or PD and 95% normal ellipses drawn for each group. A separate PCoA based on the same Bray–Curtis distance matrix was used to descriptively visualize sample distribution according to the exploratory oral-enrichment profiles.

Associations between community composition and case-control status were tested using marginal PERMANOVA with vegan::adonis2. For the primary pooled analysis, Bray–Curtis distances were modelled as “distance ~ project_id + host_status”, with marginal tests (by = "margin") and 999 permutations constrained within BioProject. The primary analysis included all 21 BioProjects (n = 3,594), and a sensitivity analysis was restricted to the 18 BioProjects containing both PD and Controls (n = 3,332). Homogeneity of multivariate dispersion between PD and Controls was assessed in the 18 BioProjects containing both groups using vegan::betadisper with distances to group spatial medians and bias adjustment, followed by permutation testing with vegan::permutest, with permutations constrained within BioProject.

Distance-based redundancy analysis was performed with vegan::dbrda to descriptively visualize the component of Bray–Curtis variation constrained by case-control status and oral-enrichment profile, after conditioning on BioProject using “Condition(project_id)”. In the main dbRDA, samples were plotted using constrained site scores and coloured by case-control status, with 95% normal ellipses drawn for controls and PD samples. Oral-profile arrows were drawn from the origin toward the centroid of each oral-enrichment profile in the constrained ordination space to aid interpretation of the compositional gradient; these arrows were not interpreted as independent statistical tests. Additional descriptive dbRDA plots were generated for all samples and separately for Controls and PD, with samples coloured by oral-enrichment profile. The full-sample model included “host_status + oral_profile + Condition(project_id)”, whereas subgroup-specific models included “oral_profile + Condition(project_id)”. Percentages shown on the dbRDA axes represent the proportion of constrained variation captured by each axis. These oral-profile ordinations were treated as descriptive visualizations rather than as independent validation of the exploratory oral-enrichment profiles.

Aitchison beta-diversity analyses were performed using SILVA-derived taxonomic count profiles. A permissive prevalence filter was applied before compositional transformation, retaining taxa detected in at least 1% of samples to remove extremely rare features while preserving low-prevalence oral-associated taxa. Zero values in the filtered count matrix were replaced using count zero multiplicative replacement with the CZM method implemented in zCompositions::cmultRepl, using frac = 0.65 and without automatic row or column deletion. The resulting compositions were centered log-ratio transformed, and Aitchison distances were calculated as Euclidean distances on the CLR-transformed matrix (3). Principal coordinates analysis (PCoA) was used to visualize unconstrained variation in Aitchison space, with separate plots generated according to case-control status and the exploratory oral-enrichment profiles.

Associations between community composition and case-control status were tested using marginal PERMANOVA with vegan::adonis2. For the primary pooled analysis, Aitchison distances were modelled as “distance ~ project_id + host_status”, with marginal tests (by = "margin") and 999 permutations constrained within BioProject. The primary analysis included all 21 BioProjects (n = 3,594), and a sensitivity analysis was restricted to the 18 BioProjects containing both PD and Controls (n = 3,332). Homogeneity of multivariate dispersion between PD and Controls was assessed in the 18 BioProjects containing both groups using vegan::betadisper with distances to group spatial medians and bias adjustment, followed by permutation testing with vegan::permutest, with permutations constrained within BioProject.

Distance-based redundancy analysis was performed using vegan::dbrda, with “host_status + oral_profile + Condition(project_id)”, to descriptively visualize the component of Aitchison variation constrained by case-control status and oral-enrichment profile after conditioning on BioProject. In the main dbRDA visualization, samples were coloured by case-control status and oral-profile centroids were projected in the constrained ordination space to aid interpretation of the compositional gradient. Additional descriptive dbRDA plots were generated for all samples and separately within PD and Control samples, using oral-enrichment profile as the main visual grouping; in subgroup analyses, case-control status was omitted because it was fixed within each subset, and BioProject was conditioned out using Condition(project_id). The full-sample model included “host_status + oral_profile + Condition(project_id)”, whereas subgroup-specific models included “oral_profile + Condition(project_id)”. Percentages shown on the dbRDA axes represent the proportion of constrained variation captured by each axis. These oral-profile ordinations were treated as descriptive visualizations rather than as independent validation of the exploratory oral-enrichment profiles.

**Sensitivity analysis after removal of oral-profile-defining taxa.** To assess whether the community structure associated with the exploratory oral-enrichment profiles was directly driven by the taxa used to define those profiles, beta-diversity analyses were repeated after removing all taxa that directly contributed to the original caries/endodontic and periodontitis scores. The original oral-enrichment profile labels were retained unchanged. Defining taxa were identified from the SILVA relative-abundance table used to construct the scores. For Bray–Curtis, these taxa were removed from the relative-abundance matrix, relative abundances were re-closed to sum to 1, and distances were recalculated. For Aitchison, the same biological taxa were mapped to the SILVA count table and removed before repeating the complete transformation workflow (1% prevalence filter, CZM zero replacement, CLR transformation and Euclidean distance calculation). In this sensitivity analysis, oral-enrichment profile was included as the factor of interest because the community distance matrices had first been recalculated after removal of all taxa used to define the original profiles. The analysis therefore tested whether the predefined profile labels remained associated with variation in the remaining microbial community, rather than retesting the profiles on the same taxa used for their construction. PCoA, marginal PERMANOVA, PERMDISP and project-conditioned dbRDA were then repeated using the recalculated distance matrices. For all samples, PERMANOVA included BioProject, case-control status and oral-enrichment profile, whereas analyses restricted to Controls or PD included BioProject and oral-enrichment profile; 999 permutations were restricted within BioProject. Multivariate dispersion among oral-enrichment profiles was assessed using distances to group spatial medians with bias adjustment. dbRDA models were conditioned on BioProject and were used descriptively to visualize residual profile-associated community structure after removal of the defining taxa. This analysis was treated as a sensitivity assessment of potential circularity and not as an independent validation of the oral-enrichment profiles.

**SUPPLEMENTARY RESULTS**

**PD-independent literature-derived oral disease panels.** All 8 caries/endodontic taxa and 18 of the 25 periodontitis taxa were represented in the pooled eHOMD dataset. In the full pooled analysis, the caries/endodontic panel showed higher relative representation in PD than in Controls (OR = 1.327, 95% CI 1.214–1.450, FDR = 8.10 × 10⁻¹⁰), whereas the periodontitis panel was not significantly associated with PD (OR = 1.035, 95% CI 0.958–1.118, FDR = 0.386). Results were very similar after restriction to the 18 BioProjects containing both PD and Control samples, excluding the three PD-only BioProjects: the caries/endodontic panel remained associated with PD (OR = 1.337, 95% CI 1.224–1.461, FDR = 2.26 × 10⁻¹⁰), whereas the periodontitis panel remained non-significant (OR = 1.038, 95% CI 0.960–1.121, FDR = 0.350) (Table S6).

When the analysis was repeated separately within the two main 16S rRNA gene regions, the caries/endodontic panel remained significantly associated with PD in both V3–V4 (OR = 1.218, 95% CI 1.083–1.370, FDR = 0.00198) and V4 datasets (OR = 1.610, 95% CI 1.389–1.864, FDR = 4.45 × 10⁻¹⁰). The periodontitis panel was not significantly associated with PD in either V3–V4 or V4 datasets (Table S6.). Although the estimated OR differed between V3–V4 and V4 there was no significant evidence after FDR correction that the PD association differed between regions (interaction FDR = 0.054).

Leave-one-taxon-out analyses showed that the caries/endodontic association was not driven by a single taxon. For the periodontitis panel, removing individual taxa did not consistently change the non-significant result.

**Sensitivity analysis after removal of oral-profile-defining taxa.** After removal of all taxa used to define the original oral-enrichment profiles and complete recalculation of the distance matrices, oral-enrichment profile remained significantly associated with variation in the remaining microbial community. Across all samples, oral-enrichment profile accounted for 1.00% of Bray–Curtis variation (R² = 0.00996, F = 14.69, FDR = 0.001) and 1.11% of Aitchison variation (R² = 0.01111, F = 17.59, FDR = 0.001). Similar associations were observed when Controls and PD samples were analysed separately (Controls: Bray–Curtis R² = 0.0139, Aitchison R² = 0.0144; PD: Bray–Curtis R² = 0.0103, Aitchison R² = 0.0109; all FDR = 0.001). Multivariate dispersion also differed among oral-enrichment profiles, including in the full-sample analyses for Bray–Curtis (F = 21.70, FDR = 0.001) and Aitchison (F = 13.22, FDR = 0.001). The PERMANOVA results were therefore interpreted cautiously and not as evidence of four discrete community states. Compared with the original ordinations (Fig. S5), removal of the profile-defining taxa led to a more apparent attenuation of profile-related structure in Bray–Curtis than in Aitchison space. Nevertheless, some profile-related organization remained visible with both distance metrics (Fig. S6).

**SUPPLEMENTARY FIGURES**

**Figure S1. Concordance of PD-associated oral taxa across MaAsLin2 and ANCOM-BC2.** Scatterplot showing −log10 FDR values from MaAsLin2 and ANCOM-BC2 for oral disease–linked taxa detected in the pooled eHOMD analysis. Dashed lines indicate FDR = 0.05 thresholds. Taxa were classified as PD-enriched or PD-depleted when both methods showed concordant effect direction and met the predefined consensus criteria. Venn-style diagrams summarize the overlap between MaAsLin2 and ANCOM-BC2 at FDR ≤ 0.10 and FDR < 0.05.

**Figure S2. Relative abundance and differential-abundance effects of oral disease–linked taxa. (A)** Distribution of log2-transformed relative abundance among samples in which each taxon was detected, shown for controls and PD samples. **(B)** Signed fold changes for PD versus controls estimated using MaAsLin2 and ANCOM-BC2. Positive values indicate PD enrichment, whereas negative values indicate PD depletion.

**Figure S3. Sensitivity of PD-versus-control differential-abundance effect estimates to read-processing strategy.** Effect estimates from the full pooled analysis were compared with those obtained after exclusion of the four datasets requiring forward-only processing (**A, C**) after restriction to paired-merged datasets only **(B, D)**, using MaAsLin2 **(A, B)** and ANCOM-BC2 **(C, D)**. Each point represents a taxon present in both the full and corresponding sensitivity analyses; taxa with FDR < 0.05 in the full analysis are highlighted. Dashed lines indicate the identity line (y = x). Spearman correlations indicate concordance between analyses.

**Figure S4. Unconstrained beta-diversity ordinations by** case-control status **and oral-enrichment profile.** Principal coordinate analysis (PCoA) of Bray–Curtis dissimilarities **(A, B)** and Aitchison distances **(C, D)** based on SILVA-derived community profiles. Samples are coloured by case-control status in panels A and C, and by oral-enrichment profile in panels B and D. Dashed lines indicate 95% normal ellipses for each group.

**Figure S5** **dbRDA of gut microbiome beta diversity using Bray–Curtis (A–D) and Aitchison (E–H) distances.** Panels A and E show the overall ordination coloured by case-control status, with arrows extending from the origin to the centroids of the four oral-enrichment profiles. Panels B and F show all samples coloured by oral-enrichment profile, while panels C and G show controls and panels D and H show PD samples. Dashed ellipses indicate 95% normal ellipses for the grouping displayed in each panel. BioProject was conditioned out in all dbRDA models. Axis percentages indicate the proportion of constrained variation represented by each dbRDA axis.

**Figure S6. dbRDA of gut microbiome beta diversity after removal of the taxa used to define the oral-enrichment profiles, using Bray–Curtis (A–D) and Aitchison (E–H) distances.** Panels A and E show the overall ordination coloured by case-control status, with arrows extending from the origin to the centroids of the four oral-enrichment profiles. Panels B and F show all samples coloured by oral-enrichment profile, while panels C and G show controls and panels D and H show PD samples. Dashed ellipses indicate 95% normal ellipses for the grouping displayed in each panel. BioProject was conditioned out in all dbRDA models. Axis percentages indicate the proportion of constrained variation represented by each dbRDA axis.

**SUPPLEMENTARY TABLES**

| **Table S1. Characteristics of the public faecal 16S rRNA datasets included in the pooled analysis.** | | | | | | | | | | |
| --- | --- | --- | --- | --- | --- | --- | --- | --- | --- | --- |
| **Dataset** | **BioProject** | **Reference** | **Countries** | **16S region** | **Platform** | **Sequencing** | **Read length** | **Mean reads** | **Samples** | |
|  |  |  |  |  |  |  |  |  | **PD** | **Control** |
| SRP326435 | PRJNA742875 | (4) | South Korea | V3-V4 | Illumina MiSeq | Paired-end | 2×300 | 37765 | 88 | 84 |
| ERP016332 | PRJEB14674 | (5) | USA | V4 | Illumina MiSeq | Single-end | 1×150 | 16948 | 197 | 130 |
| SRP337726 | PRJNA764337 | (6) | Chine | V3-V4 | Illumina MiSeq | Paired-end | 2×250 | 32904 | 106 | NA |
| SRP103472 | PRJNA381395 | (7) | Germany | V4 | Illumina MiSeq | Paired-end | 2×300 | 177552 | 26 | 38 |
| SRP367652 | PRJNA822998 | (8) | China | V3-V4 | Illumina NovaSeq | Paired-end | 2×250 | 46229 | 67 | 54 |
| SRP367653 | PRJNA822715 | (9) | China | V3-V4 | Illumina NovaSeq | Paired-end | 2×250 | 49006 | 94 | 60 |
| SRP438900 | PRJNA974928 | (10) | China | V3-V4 | Illumina NovaSeq | Paired-end | 2×250 | 53400 | 37 | 35 |
| ERP140362 | PRJEB55464 | (11) | Finland | V4 | Illumina MiSeq | Paired-end | 325/285 | 58331 | 192 | 172 |
| SRP370205 | PRJNA826738 | (12) | China | V3-V4 | Illumina MiSeq | Paired-end | 2×250 | 35348 | 118 | NA |
| SRP173877 | PRJNA510730 | (13) | Italy | V3-V4 | Illumina MiSeq | Paired-end | 2×300 | 181575 | 80 | 72 |
| SRP444507 | PRJNA975118 | (14) | Colombia | V4-V5 | Illumina MiSeq | Paired-end | 2x250 | 83192 | 25 | 25 |
| SRP502947 | PRJNA1101026 | (15) | USA | V4 | Illumina MiSeq | Paired-end | 2×250 | 48282 | 32 | 36 |
|  | DataSet_1 |  |  |  |  |  |  |  |  |  |
| SRP502947 | PRJNA1101026 | (15) | USA | V4 | Illumina HiSeq | Paired-end | 2×250 | 178416 | 89 | 62 |
|  | DataSet_2 |  |  |  |  |  |  |  |  |  |
| SRP242624 | PRJNA601994 | (16) | USA | V4 | Illumina MiSeq | Paired-end | 2×250 | 219074 | 323 | 184 |
|  | DataSet_1 |  |  |  |  |  |  |  |  |  |
| SRP242624 | PRJNA601994 | (16) | USA | V4 | Illumina MiSeq | Paired-end | 2×150 | 17044 | 201 | 132 |
|  | DataSet_2 |  |  |  |  |  |  |  |  |  |
| SRP346554 | PRJNA780943 | (17) | USA | V1-V2 | Illumina MiSeq | Paired-end | 2×250 | 34953 | 8 | 8 |
| DRP006407 | PRJDB8639 | (18) | Japan | V3-V4 | Illumina MiSeq | Paired-end | 2×300 | 104575 | 223 | 137 |
| ERP136760 | PRJEB52086 | (19) | Chine | V3-V4 | Illumina MiSeq | Paired-end | 2×300 | 14615 | 36 | 108 |
| ERP109659 | PRJEB27564 | (20) | Finland | V3-V4 | Illumina MiSeq | Paired-end | 325/285 | 88797 | 64 | 64 |
| ERP113090 | PRJEB30615 | (21) | Germany | V4-V5 | Ion Torrent PGM | Paired-end | 2x300 | 34939 | 39 | 25 |
| ERP119289 | PRJEB36138 | (22) | Italy | V3-V4 | Illumina MiSeq | Paired-end | 2x300 | 118375 | 38 | NA |
| SRP199959 | PRJNA494620 | (23) | Malaysia | V3-V4 | Illumina MiSeq | Paired-end | 2x300 | 550721 | 104 | 96 |
| SRP550468 | PRJNA1196315 | (24) | Croatia | V3-V4 | Illumina MiSeq | Paired-end | 2x300 | 93694 | 49 | 34 |
|  |  |  |  |  |  |  | **Total** | 2275735 | 2236 | 1556 |

Overview of the 23 datasets from 21 BioProjects retained for the pooled reanalysis. The table reports dataset and BioProject identifiers, original references, recruitment country, sequencing platform, read layout, read length, mean reads per sample, and the number of eligible pre-QC PD and Control samples. Final post-QC sample counts used in the pooled analyses are reported in Table S2.

**Table S2. Sequencing characteristics and dataset-specific preprocessing parameters.**

| **BioProject** | **16S rRNA target region** | **N Samples  after Qiime2** | | **Average read  (cutadapt)** | | **DADA2 processing mode** | **DADA2** | | **Primers Cutadapt/Classifier** | |
| --- | --- | --- | --- | --- | --- | --- | --- | --- | --- | --- |
|  |  |  |  |  |  |  |  |  |  |  |
|  |  | **PD** | **Control** | **Length  Forward** | **Length Reverse** |  | **truncLenF** | **truncLenR** | **Primer_F_seq** | **Primer_R_seq** |
| PRJNA742875 | V3-V4 | 88 | 84 | 234 | 230 | Paired-end | 230 | 210 | CCTACGGGNGGCWGCAG | GACTACHVGGGTATCTAATCC |
| PRJEB14674 | V4 | 200 | 130 | 150 | / | Single-end | 150 | / | GTGCCAGCMGCCGCGGTAA | GGACTACHVGGGTWTCTAAT |
| PRJNA764337 | V3-V4 | 106 | NA | 223 | 226 | Paired-end | 220 | 220 | CCTACGGGNGGCWGCAG | GGACTACHVGGGTWTCTAAT |
| PRJNA381395 | V4 | 25 | 38 | 272 | 270 | Paired-end | 272 | 220 | GTGYCAGCMGCCGCGGTAA | GACTACHVGGGTATCTAATCC |
| PRJNA822998 | V3-V4 | 67 | 54 | 224 | / | Forward-only | 223 | / | ACTCCTACGGRAGGCAGCAG | GGACTACHVGGGTWTCTAAT |
| PRJNA822715 | V3-V4 | 94 | 60 | 224 | / | Forward-only | 223 | / | ACTCCTACGGRAGGCAGCAG | GGACTACHVGGGTWTCTAAT |
| PRJNA974928 | V3-V4 | 37 | 35 | 224 | 226 | Paired-end | 223 | 223 | ACTCCTACGGRAGGCAGCAG | GGACTACHVGGGTWTCTAAT |
| PRJEB55464 | V4 | 190 | 172 | 253 | 253 | Paired-end | 252 | 204 | GTGCCAGCMGCCGCGGTAA | GGACTACHVGGGTWTCTAAT |
| PRJNA826738 | V3-V4 | 118 | NA | 221 | 226 | Paired-end | 219 | 215 | CCTACGGGNGGCWGCAG | GACTACHVGGGTATCTAATCC |
| PRJNA510730 | V3-V4 | 80 | 72 | 284 | 279 | Paired-end | 280 | 225 | CCTACGGGNGGCWGCAG | GACTACHVGGGTATCTAATCC |
| PRJNA975118 | V4-V5 | 25 | 25 | 229 | / | Forward-only | 223 | / | GTGYCAGCMGCCGCGGTAA | CCGYCAATTYMTTTRAGTTT |
| PRJNA1101026 DataSet_1 | V4 | 32 | 36 | Untrimmed | Untrimmed | Paired-end | 249 | 222 | GTGCCAGCMGCCGCGGTAA | GGACTACNVGGGTWTCTAAT |
| PRJNA1101026 DataSet_2 | V4 | 89 | 62 | Untrimmed | Untrimmed | Paired-end | 249 | 133 | GTGCCAGCMGCCGCGGTAA | GGACTACNVGGGTWTCTAAT |
| PRJNA601994 DataSet_1 | V4 | 323 | 184 | 232 | 231 | Paired-end | 230 | 222 | GTGCCAGCMGCCGCGGTAA | GGACTACNVGGGTWTCTAAT |
| PRJNA601994 DataSet_2 | V4 | 192 | 127 | 130 | 130 | Paired-end | 129 | 127 | GTGCCAGCMGCCGCGGTAA | GGACTACNVGGGTWTCTAAT |
| PRJNA780943 | V1-V2 | 8 | 8 | 229 | 229 | Paired-end | 224 | 219 | AGAGTTTGATCMTGGCTCAG | TGCTGCCTCCCGTAGGAGT |
| PRJDB8639 | V3-V4 | 223 | 137 | 334 | 230 | Paired-end | 260 | 193 | CCTACGGGNGGCWGCAG | GACTACHVGGGTATCTAATCC |
| PRJEB52086 | V3-V4 | 19 | 50 | 284 | 281 | Paired-end | 280 | 230 | CCTACGGGNGGCWGCAG | GGACTACNVGGGTWTCTAAT |
| PRJEB27564 | V3-V4 | 61 | 63 | 307 | 262 | Paired-end | 300 | 205 | CCTACGGGNGGCWGCAG | GACTACHVGGGTATCTAATCC |
| PRJEB30615 | V4-V5 | 38 | 25 | 284 | 279 | Paired-end | 284 | 217 | AYTGGGYDTAAAGNG | CCGTCAATTCMTTTRAGTTT |
| PRJEB36138 | V3-V4 | 38 | NA | 284 | / | Forward-only | 255 | / | CCTACGGGNGGCWGCAG | GACTACHVGGGTATCTAATCC |
| PRJNA494620 | V3-V4 | 52 | 44 | 233 | 229 | Paired-end | 233 | 220 | CCTACGGGNGGCWGCAG | GACTACHVGGGTATCTAATCC |
| PRJNA1196315 | V3-V4 | 49 | 34 | 284 | 281 | Paired-end | 280 | 222 | CCTACGGGNGGCWGCAG | GGACTACHVGGGTWTCTAAT |
| **Total** | | 2154 | 1440 |  |  | |  |  | |  |

16S rRNA target regions and preprocessing settings for the 23 datasets included in the pooled analysis. The table reports BioProject accession, target region, post-QC sample counts, read lengths, DADA2 processing mode and truncation settings, and primer sequences. “Forward-only” denotes datasets originally generated as paired-end but processed using forward reads only because reverse-read quality and post-truncation overlap did not permit robust merging; “Single-end” denotes the original sequencing design.

**Table S3.** **Differential abundance and descriptive summaries of oral disease–linked taxa in the pooled eHOMD analysis.**

| **Taxa** | **MaAslin2** | |  | **ANCOM-BC2** | |  | **Prevalence (%)** | |  | **Mean** | | | | | **References for association with dental diseases** |
| --- | --- | --- | --- | --- | --- | --- | --- | --- | --- | --- | --- | --- | --- | --- | --- |
|  | **Effect size (β)** | **FDR** |  | **logFC** | **FDR** |  | **PD** | **Controls** |  | **PD** | **± SD** |  | **Controls** | **± SD** |  |
| **Taxa associated with dental caries** | | | | | | | | | | | | | | | |
| *Bifidobacterium* | 1.241 | 3.56E-08 |  | 0.434 | 3.66E-04 |  | 71.03% | 67.43% |  | 2.04E-02 | 4.67E-02 |  | 8.97E-03 | 2.45E-02 | (25) |
| *Lactobacillus* | 0.544 | 4.68E-08 |  | 0.49 | 3.08E-02 |  | 16.76% | 13.06% |  | 9.08E-04 | 7.86E-03 |  | 2.46E-04 | 2.26E-03 | (26) |
| *Veillonella atypica* | -0.276 | 2.44E-03 |  | -0.683 | 1.32E-03 |  | 9.56% | 13.06% |  | 1.94E-04 | 1.62E-03 |  | 4.80E-04 | 2.95E-03 | (27) |
| *Veillonella* | -0.469 | 8.52E-03 |  | -0.493 | 1.57E-04 |  | 37.09% | 43.68% |  | 9.95E-04 | 5.58E-03 |  | 1.67E-03 | 7.77E-03 | (27) |
| *Streptococcus mutans* | 1.045 | 4.44E-25 |  | 0.283 | 1.37E-01 |  | 24.61% | 12.85% |  | 4.69E-04 | 4.66E-03 |  | 1.75E-04 | 2.33E-03 | (28) |
| *Eggerthia catenaformis* | 0.803 | 1.95E-07 |  | 0.014 | 9.68E-01 |  | 45.50% | 41.32% |  | 1.96E-03 | 8.03E-03 |  | 1.61E-03 | 6.40E-03 | (27) |
| *Streptococcus anginosus* | 0.43 | 4.97E-07 |  | -0.05 | 8.85E-01 |  | 18.15% | 12.78% |  | 3.04E-04 | 2.03E-03 |  | 1.38E-04 | 8.15E-04 | (29) |
| *Bifidobacterium subtile* | 0.583 | 1.05E-06 |  | 0.279 | 2.56E-01 |  | 16.90% | 10.90% |  | 6.44E-04 | 3.14E-03 |  | 2.38E-04 | 1.34E-03 | (30) |
| *Bifidobacterium dentium* | 0.668 | 1.09E-05 |  | 0.029 | 9.39E-01 |  | 42.62% | 34.17% |  | 1.02E-02 | 3.73E-02 |  | 5.71E-03 | 1.94E-02 | (31) |
| *Finegoldia magna* | 0.399 | 9.14E-05 |  | 0.113 | 6.86E-01 |  | 18.20% | 13.40% |  | 3.84E-04 | 2.82E-03 |  | 1.40E-04 | 8.97E-04 | (32) |
| *Pseudoramibacter alactolyticus* | 0.388 | 7.78E-04 |  | -0.066 | 7.26E-01 |  | 31.80% | 26.53% |  | 7.51E-04 | 5.99E-03 |  | 7.08E-04 | 6.15E-03 | (33) |
| *Bifidobacterium longum* | 0.352 | 2.57E-03 |  | 0.154 | 3.81E-01 |  | 38.25% | 33.96% |  | 7.61E-03 | 2.80E-02 |  | 3.13E-03 | 1.48E-02 | (34) |
| *Schaalia* | 0.289 | 5.13E-03 |  | -0.08 | 6.02E-01 |  | 33.66% | 31.39% |  | 3.31E-04 | 1.22E-03 |  | 2.62E-04 | 1.57E-03 | (35) |
| *Olsenella sp. HMT-939* | 0.388 | 3.02E-02 |  | 0.023 | 9.27E-01 |  | 73.54% | 73.75% |  | 8.35E-03 | 1.94E-02 |  | 5.83E-03 | 1.30E-02 | (36) |
| *Olsenella* | 0.266 | 8.96E-02 |  | -0.008 | 9.94E-01 |  | 29.39% | 29.51% |  | 8.44E-04 | 9.35E-03 |  | 4.20E-04 | 1.68E-03 | (37) |
| *Veillonella parvula* | 0.217 | 2.57E-01 |  | -0.365 | 3.94E-02 |  | 22.05% | 20.28% |  | 9.15E-04 | 9.26E-03 |  | 1.06E-03 | 9.14E-03 | (38) |
| **Taxa associated with periodontitis** | | | | | | | | | | | | | | | |
| *Lachnoanaerobaculum* | -0.63 | 5.80E-14 |  | -0.526 | 8.84E-09 |  | 75.81% | 81.18% |  | 1.40E-02 | 2.71E-02 |  | 2.09E-02 | 4.01E-02 | (39) |
| *Granulicatella adiacens* | -0.11 | 3.48E-01 |  | -0.272 | 3.48E-02 |  | 18.85% | 22.08% |  | 7.74E-05 | 3.96E-04 |  | 9.77E-05 | 4.73E-04 | (40) |
| *Mogibacterium* | 0.733 | 5.34E-08 |  | 0.228 | 5.07E-02 |  | 44.48% | 41.39% |  | 6.12E-04 | 2.16E-03 |  | 3.34E-04 | 1.21E-03 | (41) |
| *Methanobrevibacter oralis* | 0.758 | 7.50E-06 |  | 0.558 | 5.27E-04 |  | 32.92% | 31.04% |  | 3.49E-03 | 1.59E-02 |  | 1.66E-03 | 1.04E-02 | (42) |
| *Desulfovibrio fairfieldensis* | 0.867 | 7.18E-08 |  | 0.235 | 1.14E-01 |  | 36.77% | 34.10% |  | 1.37E-03 | 4.10E-03 |  | 1.10E-03 | 3.78E-03 | (43) |
| *Hoylesella buccalis* | 0.542 | 7.18E-08 |  | 0.289 | 2.35E-01 |  | 16.20% | 9.86% |  | 6.49E-04 | 7.88E-03 |  | 1.24E-04 | 1.40E-03 | (44) |
| *Tannerella* | 0.455 | 5.81E-04 |  | 0.054 | 8.77E-01 |  | 24.33% | 20.21% |  | 5.47E-04 | 2.74E-03 |  | 2.53E-04 | 1.23E-03 | (45) |
| *Megasphaera micronuciformis* | 0.307 | 2.43E-02 |  | -0.011 | 9.94E-01 |  | 18.85% | 18.26% |  | 3.31E-03 | 2.03E-02 |  | 2.60E-03 | 1.38E-02 | (46) |
| *Selenomonas sp. HMT-442* | 0.173 | 2.61E-02 |  | 0.151 | 3.81E-01 |  | 10.49% | 10.28% |  | 3.69E-05 | 2.10E-04 |  | 3.91E-05 | 3.64E-04 | (47) |
| *Desulfovibrio* | 0.286 | 4.81E-02 |  | 0.037 | 8.86E-01 |  | 32.82% | 32.36% |  | 1.17E-03 | 4.59E-03 |  | 8.91E-04 | 3.07E-03 | (48) |
| *Selenomonas* | 0.372 | 5.87E-02 |  | -0.21 | 1.22E-01 |  | 71.40% | 74.58% |  | 1.62E-02 | 3.32E-02 |  | 1.58E-02 | 3.00E-02 | (49) |
| *Dialister invisus* | 0.168 | 5.78E-01 |  | -0.432 | 5.10E-03 |  | 41.46% | 40.28% |  | 5.36E-03 | 1.86E-02 |  | 5.26E-03 | 1.39E-02 | (50) |
| *Rothia mucilaginosa* | -0.077 | 6.04E-01 |  | -0.401 | 1.45E-03 |  | 22.75% | 21.18% |  | 1.48E-04 | 8.08E-04 |  | 1.01E-04 | 5.57E-04 | (51) |
| *Lautropia dentalis* | -0.055 | 8.35E-01 |  | -0.329 | 1.83E-02 |  | 42.34% | 48.75% |  | 2.16E-03 | 6.34E-03 |  | 2.57E-03 | 7.11E-03 | (52) |
| *Alloprevotella* | 0.037 | 9.14E-01 |  | -0.533 | 3.24E-03 |  | 26.18% | 27.15% |  | 2.04E-03 | 8.12E-03 |  | 2.34E-03 | 9.42E-03 | (53) |
| *Alloprevotella sp. HMT-308* | -0.006 | 9.75E-01 |  | -0.527 | 1.05E-02 |  | 11.14% | 11.88% |  | 7.35E-04 | 3.73E-03 |  | 9.07E-04 | 3.98E-03 | (54) |

Oral disease annotation indicates classification as caries/endodontic-associated or periodontitis/subgingival plaque-associated based on eHOMD annotation and supporting literature. MaAsLin2 coefficients and ANCOM-BC2 log-fold-change estimates correspond to the PD-versus-Control contrast; positive values indicate higher abundance in PD and negative values lower abundance in PD. Corresponding BH FDR-adjusted q-values are reported for both methods. Prevalence indicates the percentage of samples in which each taxon was detected, and mean abundance is reported as relative abundance ± standard deviation for PD and Controls. Taxa were retained when significantly associated with case-control status (FDR < 0.05) in at least one differential-abundance method.

**Table S4. Robustness of PD-associated differential-abundance results to read-processing strategy.**

| **Sensitivity analysis** | **N Samples** | **Method** | **Spearman ρ, full vs sensitivity** | **Direction concordance, n/N** | **FDR < 0.05 retained, n/N** |
| --- | --- | --- | --- | --- | --- |
| Exclusion of four forward-only datasets | 3,231 | MaAsLin2 | 0.97 | 25/25 | 22/25 |
| Exclusion of four forward-only datasets | 3,231 | ANCOM-BC2 | 0.97 | 13/13 | 12/13 |
| Paired-merged datasets only | 2,901 | MaAsLin2 | 0.95 | 26/26 | 23/26 |
| Paired-merged datasets only | 2,901 | ANCOM-BC2 | 0.97 | 14/14 | 11/14 |

Spearman correlations were calculated across all taxa available in both the primary full analysis and the corresponding sensitivity analysis. For the two concordance columns, the denominator represents the number of taxa that were significant (FDR < 0.05) in the primary analysis and were also retained in the sensitivity model. The numerator represents the number of these taxa that retained the same effect direction or remained significant at FDR < 0.05, respectively. Thus, for example, 22/25 indicates that 22 of the 25 taxa significant in the primary MaAsLin2 analysis remained significant after exclusion of the four forward-only datasets.

**Table S5. Case-control status and amplicon-region association patterns for oral disease–linked taxa in the pooled MaAsLin2 analysis.**

| **MaAsLin2** | | | | | | |
| --- | --- | --- | --- | --- | --- | --- |
| **Taxa** | **PD effect size (β)** | **PD FDR** | **Selected region contrast** | **Region effect size (β)** | **Region FDR** | **Association pattern** |
| **Taxa associated with dental caries/endodontic disease** | | | | | | |
| *Bifidobacterium* | 1.24 | 3.56E-08 | V4 > V3–V4 | 4.27 | 5.87E-02 | PD-associated |
| *Lactobacillus* | 0.54 | 4.68E-08 | V4 < V3–V4 | −0.64 | 4.92E-01 | PD-associated |
| *Limosilactobacillus* | 0.68 | 2.15E-07 | V4-V5 > V3-V4 | 1.69 | 1.84E-01 | PD-associated |
| *Veillonella atypica* | -0.28 | 2.44E-03 | V4 < V3-V4 | -1.57 | 9.81E-03 | Mixed PD/region-associated |
| *Veillonella* | -0.47 | 8.52E-03 | V1-V2 < V3-V4 | -1.08 | 8.16E-01 | PD-associated |
| *Streptococcus mutans* | 1.04 | 4.44E-25 | V4-V5 < V3-V4 | 3.62 | 1.90E-01 | PD-associated |
| *Eggerthia catenaformis* | 0.8 | 1.95E-07 | V1-V2 < V3-V4 | -2.88 | 5.08E-01 | PD-associated |
| *Streptococcus anginosus* | 0.43 | 4.97E-07 | V4-V5 > V3-V4 | 0.8 | 3.99E-01 | PD-associated |
| *Bifidobacterium subtile* | 0.58 | 1.05E-06 | V4 < V3–V4 | -1.49 | 9.37E-03 | Mixed PD/region-associated |
| *Bifidobacterium dentium* | 0.67 | 1.09E-05 | V4 < V3-V4 | -5.55 | 1.21E-04 | Mixed PD/region-associated |
| *Finegoldia magna* | 0.4 | 9.14E-05 | V4 > V3-V4 | 1.17 | 3.83E-02 | Mixed PD/region-associated |
| *Pseudoramibacter alactolyticus* | 0.39 | 7.78E-04 | V4–V5 > V3–V4 | 1.79 | 6.31E-01 | PD-associated |
| *Bifidobacterium longum* | 0.35 | 2.57E-03 | V4 < V3–V4 | -6.26 | 3.56E-08 | Mixed PD/region-associated |
| *Schaalia* | 0.29 | 5.13E-03 | V4-V5 > V3-V4 | 2.88 | 1.84E-01 | PD-associated |
| *Olsenella sp. HMT-939* | 0.39 | 3.02E-02 | V4-V5 > V3-V4 | 4.61 | 1.14E-01 | PD-associated |
| *Olsenella* | 0.27 | 8.96E-02 | V4-V5 > V3-V4 | 4.01 | 9.23E-03 | Region-associated |
| **Taxa associated with periodontitis/subgingival plaque** | | | | | | |
| *Lachnoanaerobaculum* | -0.63 | 5.80E-14 | V1-V2 < V3-V4 | -6.29 | 3.06E-01 | PD-associated |
| *Mogibacterium* | 0.73 | 5.34E-08 | V4–V5 > V3–V4 | 1.74 | 4.98E-01 | PD-associated |
| *Methanobrevibacter oralis* | 0.76 | 7.50E-06 | V4 > V3–V4 | 2.3 | 1.82E-01 | PD-associated |
| *Desulfovibrio fairfieldensis* | 0.87 | 7.18E-08 | V4 > V3-V4 | 1.59 | 2.44E-01 | PD-associated |
| *Hoylesella buccalis* | 0.54 | 7.18E-08 | V4 > V3-V4 | 0.99 | 7.07E-02 | PD-associated |
| *Tannerella* | 0.46 | 5.81E-04 | V1-V2 > V3-V4 | 5.47 | 1.65E-02 | Mixed PD/region-associated |
| *Megasphaera micronuciformis* | 0.31 | 2.43E-02 | V4 < V3–V4 | −2.59 | 2.16E-01 | PD-associated |
| *Selenomonas sp. HMT-442* | 0.17 | 2.61E-02 | V4 > V3-V4 | 1.19 | 1.27E-02 | Mixed PD/region-associated |
| *Desulfovibrio* | 0.29 | 4.81E-02 | V4 > V3-V4 | 3.41 | 5.06E-02 | PD-associated |
| *Alloprevotella sp. HMT-308* | -0.01 | 9.75E-01 | V4 < V3–V4 | -1.95 | 4.20E-02 | Region-associated |

The table summarizes MaAsLin2 associations of oral disease–associated taxa with case-control status and 16S rRNA gene amplicon region. Controls were used as the reference category for case-control status and V3–V4 as the reference level for amplicon region. Positive PD coefficients indicate higher abundance in PD, whereas negative coefficients indicate lower abundance. For each taxon, the region contrast with the lowest FDR-adjusted P value was selected, and the reported region coefficient and Region FDR correspond to that same contrast. Taxa were classified as PD-associated, region-associated, or mixed PD/region-associated according to the criteria described in the Supplementary Methods.

**Table S6. Composition and PD association of the PD-independent literature-derived oral disease-associated panels.**

| **A. Literature-derived panel composition** | | | |  |  |
| --- | --- | --- | --- | --- | --- |
| **Literature taxon** | **Main supporting reference(s)** | **Represented in pooled eHOMD** | **Included in analysis** |  |  |
| **Caries/endodontic-associated panel** | | | |  |  |
| *Streptococcus mutans* | (55) | Yes | Yes |  |  |
| *Scardovia wiggsiae* | (56) | Yes | Yes |  |  |
| *Streptococcus sobrinus* | (57) | Yes | Yes |  |  |
| *Bifidobacterium dentium* | (58) | Yes | Yes |  |  |
| *Selenomonas sputigena* | (59) | Yes | Yes |  |  |
| *Pseudoramibacter alactolyticus* | (60) | Yes | Yes |  |  |
| *Porphyromonas endodontalis* | (60) | Yes | Yes |  |  |
| *Olsenella uli* | (60) | Yes | Yes |  |  |
| **Periodontitis-associated panel** | | | |  |  |
| *Eubacterium brachy* | (61) | No | No |  |  |
| *Treponema lecithinolyticum* | (61) | No | No |  |  |
| *Treponema medium* | (61) | No | No |  |  |
| *Fusobacterium nucleatum subsp. vincentii* | (61) | Yes | Yes |  |  |
| *Porphyromonas gingivalis* | (61) | Yes | Yes |  |  |
| *Streptococcus constellatus* | (61) | Yes | Yes |  |  |
| *Treponema socranskii* | (61) | No | No |  |  |
| *Filifactor alocis* | (61) | Yes | Yes |  |  |
| *Eubacterium nodatum* | (61) | Yes | Yes |  |  |
| *Eubacterium saphenum* | (61) | No | No |  |  |
| *Treponema maltophilum* | (61) | No | No |  |  |
| *Tannerella forsythia* | (61) | Yes | Yes |  |  |
| *Fretibacterium fastidiosum* | (61) | Yes | Yes |  |  |
| *Mogibacterium timidum* | (61) | Yes | Yes |  |  |
| *Treponema denticola* | (61) | Yes | Yes |  |  |
| *Fusobacterium periodonticum* | (62) | Yes | Yes |  |  |
| *Fusobacterium nucleatum sensu stricto* | (62) | Yes | Yes |  |  |
| *Campylobacter showae* | (62) | Yes | Yes |  |  |
| *Campylobacter rectus* | (62) | Yes | Yes |  |  |
| *Prevotella intermedia* | (62) | Yes | Yes |  |  |
| *Parvimonas micra* | (62) | Yes | Yes |  |  |
| *Dialister invisus* | (63) | Yes | Yes |  |  |
| *Peptostreptococcus stomatis* | (63) | Yes | Yes |  |  |
| *Aggregatibacter actinomycetemcomitans* | (63) | No | No |  |  |
| *Desulfobulbus oralis* | (64) | Yes | Yes |  |  |
|  |  |  |  |  |  |
|  |  |  |  |  |  |
| **B. Panel-level association with PD** | | | | | |
| **Panel** | **Analysis** | **n** | **OR  (PD vs Controls)** | **95% CI** | **FDR** |
| Caries/endodontic | Full pooled | 3594 | 1.327 | 1.214–1.450 | 8.10E-10 |
| Caries/endodontic | Case-control projects only | 3332 | 1.337 | 1.224–1.461 | 2.26E-10 |
| Caries/endodontic | V3-V4 only | 1665 | 1.218 | 1.083–1.370 | 2.00E-03 |
| Caries/endodontic | V4 only | 1800 | 1.610 | 1.389–1.864 | 4.45E-10 |
| Periodontitis | Full pooled | 3594 | 1.035 | 0.958–1.118 | 3.86E-01 |
| Periodontitis | Case-control projects only | 3332 | 1.038 | 0.960–1.121 | 3.50E-01 |
| Periodontitis | V3-V4 only | 1665 | 1.085 | 0.962–1.224 | 1.85E-01 |
| Periodontitis | V4 only | 1800 | 1.017 | 0.917–1.129 | 7.46E-01 |

Literature-derived panels were defined independently of PD association testing and subsequently intersected with taxa represented in the pooled eHOMD dataset. ORs compare the relative representation of panel-assigned reads in PD versus Controls; OR > 1 indicates higher relative representation in PD. Models were adjusted for 16S rRNA gene region and included BioProject as a random intercept. Taxa not represented in eHOMD remained part of the literature-defined panel but were excluded from quantitative analysis.

**Table S7. Beta-diversity analyses of case-control status.**

| A. PERMANOVA — case-control status | | | | | | | | |
| --- | --- | --- | --- | --- | --- | --- | --- | --- |
| **Distance** | **Dataset** | **n** | | **BioProjects** | **Case-control R²** | **F** | **P** | **BioProject marginal R²** |
| Bray–Curtis | All projects | | 3,594 | 21 | 0.00432 | 18.86 | 0.001 | 0.17433 |
| Bray–Curtis | Mixed projects only | | 3,332 | 18 | 0.00479 | 18.92 | 0.001 | 0.15465 |
| Aitchison | All projects | | 3,594 | 21 | 0.00217 | 10.28 | 0.001 | 0.24329 |
| Aitchison | Mixed projects only | | 3,332 | 18 | 0.00230 | 10.05 | 0.001 | 0.23692 |
|  |  | |  |  |  |  |  |  |
| B. PERMDISP — PD vs Controls in the 18 mixed BioProjects | | | | | | |  |  |
| **Distance** | **n** | | **Controls** | **PD** | **F** | **P** |  |  |
| Bray–Curtis | 3,332 | | 0.5477 | 0.5677 | 79.36 | 0.001 |  |  |
| Aitchison | 3,332 | | 33.188 | 34.725 | 92.83 | 0.001 |  |  |
|  |  | |  |  |  |  |  |  |

**(A)** Marginal PERMANOVA results for the association between case-control status and community composition in the full dataset (21 BioProjects, including three PD-only projects) and in a sensitivity analysis restricted to the 18 BioProjects containing both PD and Controls. BioProject marginal R² values are shown for comparison. **(B)** PERMDISP results comparing multivariate dispersion between PD and Controls within the 18 mixed BioProjects. Mean distances to the group spatial median are reported for Controls and PD for each distance metric.

**Table S8. Concordance of eHOMD-derived labels with NCBI BLASTn annotations**.

The table summarizes, for each taxon within the oral disease-associated categories (dental caries or periodontitis), the prevalence (number of samples, n), relative abundance (%), and standard deviation calculated across all analysed studies using either eHOMD- or SILVA-based taxonomic labels. BLASTn analyses were performed on sequences from a single analysed study to assess the consistency of eHOMD-derived assignments with NCBI annotations, based on the top 100 bacterial hits (concordance, %). A second targeted (“forced”) BLASTn analysis was additionally carried out by restricting the search to the taxon assigned by eHOMD.

**REFERENCE**

1.	Oksanen J, Simpson GL, Blanchet FG, et al. vegan: Community Ecology Package. 2001;2.7-2. https://doi.org/10.32614/CRAN.package.vegan

2.	Bray JR, Curtis JT. An Ordination of the Upland Forest Communities of Southern Wisconsin. Ecol Monogr 1957;27(4):325–349. https://doi.org/10.2307/1942268

3.	Aitchison J, Greenacre M. Biplots of Compositional Data. J R Stat Soc Ser C Appl Stat 2002;51(4):375–392. https://doi.org/10.1111/1467-9876.00275

4.	Jo S, Kang W, Hwang YS, et al. Oral and gut dysbiosis leads to functional alterations in Parkinson’s disease. Npj Park Dis 2022;8(1):87. https://doi.org/10.1038/s41531-022-00351-6

5.	Hill-Burns EM, Debelius JW, Morton JT, et al. Parkinson’s Disease and PD Medications Have Distinct Signatures of the Gut Microbiome. Mov Disord Off J Mov Disord Soc 2017;32(5):739–749. https://doi.org/10.1002/mds.26942

6.	Zhang Y, He X, Qian Y, et al. Plasma branched-chain and aromatic amino acids correlate with the gut microbiota and severity of Parkinson’s disease. Npj Park Dis 2022;8(1):48. https://doi.org/10.1038/s41531-022-00312-z

7.	Heintz-Buschart A, Pandey U, Wicke T, et al. The nasal and gut microbiome in Parkinson’s disease and idiopathic rapid eye movement sleep behavior disorder. Mov Disord Off J Mov Disord Soc 2018;33(1):88–98. https://doi.org/10.1002/mds.27105

8.	Zhang P, Huang P, Du J, et al. Specific gut microbiota alterations in essential tremor and its difference from Parkinson’s disease. NPJ Park Dis 2022;8(1):98. https://doi.org/10.1038/s41531-022-00359-y

9.	Zhang P, Huang P, Li Y, et al. Relationships Between Rapid Eye Movement Sleep Behavior Disorder and Parkinson’s Disease: Indication from Gut Microbiota Alterations. Aging Dis 2024;15(1):357–368. https://doi.org/10.14336/AD.2023.0518

10.	Huang P, Zhang P, Du J, et al. Association of fecal short-chain fatty acids with clinical severity and gut microbiota in essential tremor and its difference from Parkinson’s disease. NPJ Park Dis 2023;9(1):115. https://doi.org/10.1038/s41531-023-00554-5

11.	Boertien JM, Murtomäki K, Pereira PAB, et al. Fecal microbiome alterations in treatment-naive de novo Parkinson’s disease. NPJ Park Dis 2022;8(1):129. https://doi.org/10.1038/s41531-022-00395-8

12.	Yang X, He X, Xu S, et al. Effect of Lacticaseibacillus paracasei strain Shirota supplementation on clinical responses and gut microbiome in Parkinson’s disease. Food Funct 2023;14(15):6828–6839. https://doi.org/10.1039/D3FO00728F

13.	Pietrucci D, Cerroni R, Unida V, et al. Dysbiosis of gut microbiota in a selected population of Parkinson’s patients. Parkinsonism Relat Disord 2019;65:124–130. https://doi.org/10.1016/j.parkreldis.2019.06.003

14.	Forero-Rodríguez J, Zimmermann J, Taubenheim J, et al. Changes in Bacterial Gut Composition in Parkinson’s Disease and Their Metabolic Contribution to Disease Development: A Gut Community Reconstruction Approach. Microorganisms 2024;12(2):325. https://doi.org/10.3390/microorganisms12020325

15.	Kwon D, Zhang K, Paul KC, et al. Diet and the gut microbiome in patients with Parkinson’s disease. NPJ Park Dis 2024;10(1):89. https://doi.org/10.1038/s41531-024-00681-7

16.	Wallen ZD, Appah M, Dean MN, et al. Characterizing dysbiosis of gut microbiome in PD: evidence for overabundance of opportunistic pathogens. Npj Park Dis 2020;6(1):11. https://doi.org/10.1038/s41531-020-0112-6

17.	Rusch C, Beke M, Tucciarone L, et al. Mediterranean Diet Adherence in People With Parkinson’s Disease Reduces Constipation Symptoms and Changes Fecal Microbiota After a 5-Week Single-Arm Pilot Study. Front Neurol 2021;12:794640. https://doi.org/10.3389/fneur.2021.794640

18.	Nishiwaki H, Ito M, Hamaguchi T, et al. Short chain fatty acids-producing and mucin-degrading intestinal bacteria predict the progression of early Parkinson’s disease. NPJ Park Dis 2022;8(1):65. https://doi.org/10.1038/s41531-022-00328-5

19.	Huang B, Chau SWH, Liu Y, et al. Gut microbiome dysbiosis across early Parkinson’s disease, REM sleep behavior disorder and their first-degree relatives. Nat Commun 2023;14(1):2501. https://doi.org/10.1038/s41467-023-38248-4

20.	Aho VTE, Pereira PAB, Voutilainen S, et al. Gut microbiota in Parkinson’s disease: Temporal stability and relations to disease progression. EBioMedicine 2019;44:691–707. https://doi.org/10.1016/j.ebiom.2019.05.064

21.	Weis S, Schwiertz A, Unger MM, et al. Effect of Parkinson’s disease and related medications on the composition of the fecal bacterial microbiota. NPJ Park Dis 2019;5:28. https://doi.org/10.1038/s41531-019-0100-x

22.	Vascellari S, Melis M, Cossu G, et al. Genetic variants of TAS2R38 bitter taste receptor associate with distinct gut microbiota traits in Parkinson’s disease: A pilot study. Int J Biol Macromol 2020;165(Pt A):665–674. https://doi.org/10.1016/j.ijbiomac.2020.09.056

23.	Tan AH, Chong CW, Lim S-Y, et al. Gut Microbial Ecosystem in Parkinson Disease: New Clinicobiological Insights from Multi-Omics. Ann Neurol 2021;89(3):546–559. https://doi.org/10.1002/ana.25982

24.	Papić E, Rački V, Hero M, et al. Microbial diversity in drug-naïve Parkinson’s disease patients. PloS One 2025;20(8):e0328761. https://doi.org/10.1371/journal.pone.0328761

25.	Manome A, Abiko Y, Kawashima J, et al. Acidogenic Potential of Oral Bifidobacterium and Its High Fluoride Tolerance. Front Microbiol 2019;10:1099. https://doi.org/10.3389/fmicb.2019.01099

26.	Caufield PW, Schön CN, Saraithong P, Li Y, Argimón S. Oral Lactobacilli and Dental Caries. J Dent Res 2015;94(9 Suppl):110S-118S. https://doi.org/10.1177/0022034515576052

27.	Zhou P, Manoil D, Belibasakis GN, Kotsakis GA. Veillonellae: Beyond Bridging Species in Oral Biofilm Ecology. Front Oral Health 2021;2:774115. https://doi.org/10.3389/froh.2021.774115

28.	Mazurel D, Brandt BW, Boomsma M, et al. Streptococcus mutans and Caries: A Systematic Review and Meta-Analysis. J Dent Res 2025;104(6):594–603. https://doi.org/10.1177/00220345241303880

29.	Furuholm J, Uittamo J, Rautaporras N, Välimaa H, Snäll J. Streptococcus anginosus: a stealthy villain in deep odontogenic abscesses. Odontology 2023;111(2):522–530. https://doi.org/10.1007/s10266-022-00763-z

30.	Mantzourani M, Fenlon M, Beighton D. Association between Bifidobacteriaceae and the clinical severity of root caries lesions. Oral Microbiol Immunol 2009;24(1):32–37. https://doi.org/10.1111/j.1399-302X.2008.00470.x

31.	Toh H, Hayashi J, Oshima K, et al. Complete Genome Sequence of Bifidobacterium dentium Strain JCM 1195T, Isolated from Human Dental Caries. Genome Announc 2015;3(2):10.1128/genomea.00284-15. https://doi.org/10.1128/genomea.00284-15

32.	Siqueira JF, Rôças IN. *Pseudoramibacter alactolyticus* in Primary Endodontic Infections. J Endod 2003;29(11):735–738. https://doi.org/10.1097/00004770-200311000-00012

33.	Siqueira JF, Rôças IN. Microbiology and Treatment of Acute Apical Abscesses. Clin Microbiol Rev 2013;26(2):255–273. https://doi.org/10.1128/CMR.00082-12

34.	Nakajo K, Takahashi N, Beighton D. Resistance to acidic environments of caries-associated bacteria: Bifidobacterium dentium and Bifidobacterium longum. Caries Res 2010;44(5):431–437. https://doi.org/10.1159/000318582

35.	Jang E-Y, Chun J, Kwack KH, Moon J-H, Lee J-H. Complete genome sequence of Schaalia odontolytica isolated from subgingival biofilm. BMC Genomic Data 2024;25(1):15. https://doi.org/10.1186/s12863-023-01184-9

36.	Nardello LCL, Amado PPP, Franco DC, et al. Next-Generation Sequencing to Assess Potentially Active Bacteria in Endodontic Infections. J Endod 2020;46(8):1105–1112. https://doi.org/10.1016/j.joen.2020.05.004

37.	Rôças IN, Alves FRF, Rachid CTCC, et al. Microbiome of Deep Dentinal Caries Lesions in Teeth with Symptomatic Irreversible Pulpitis. PLOS ONE 2016;11(5):e0154653. https://doi.org/10.1371/journal.pone.0154653

38.	Wei Y, Zhang Y, Zhuang Y, et al. Veillonella parvula acts as a pathobiont promoting the biofilm virulence and cariogenicity of Streptococcus mutans in adult severe caries. Microbiol Spectr;12(11):e04318-23. https://doi.org/10.1128/spectrum.04318-23

39.	Lim YK, Park S-N, Jo E, et al. Lachnoanaerobaculum gingivalis sp. nov., Isolated from Human Subgingival Dental Plaque of a Gingivitis Lesion. Curr Microbiol 2019;76(10):1147–1151. https://doi.org/10.1007/s00284-019-01747-z

40.	Siqueira JF, Rôças IN. *Catonella morbi* and *Granulicatella adiacens*: new species in endodontic infections. Oral Surg Oral Med Oral Pathol Oral Radiol Endodontology 2006;102(2):259–264. https://doi.org/10.1016/j.tripleo.2005.09.021

41.	Casarin RCV, Saito D, Santos VR, et al. Detection of Mogibacterium timidum in subgingival biofilm of aggressive and non-diabetic and diabetic chronic periodontitis patients. Braz J Microbiol 2012;43(3):931–937. https://doi.org/10.1590/S1517-838220120003000012

42.	Pilliol V, Mahmoud Abdelwadoud B, Aïcha H, et al. Methanobrevibacter oralis: a comprehensive review. J Oral Microbiol;16(1):2415734. https://doi.org/10.1080/20002297.2024.2415734

43.	Loubinoux J, Bisson-Boutelliez C, Miller N, Le Faou AE. Isolation of the provisionally named Desulfovibrio fairfieldensis from human periodontal pockets. Oral Microbiol Immunol 2002;17(5):321–323. https://doi.org/10.1034/j.1399-302X.2002.170510.x

44.	Ovsepian A, Kardaras FS, Skoulakis A, Hatzigeorgiou AG. Microbial signatures in human periodontal disease: a metatranscriptome meta-analysis. Front Microbiol;15. https://doi.org/10.3389/fmicb.2024.1383404

45.	Mohanty R, Asopa SJ, Joseph MD, et al. Red complex: Polymicrobial conglomerate in oral flora: A review. J Fam Med Prim Care 2019;8(11):3480–3486. https://doi.org/10.4103/jfmpc.jfmpc_759_19

46.	Nallabelli N, Patil PP, Pal VK, et al. Biochemical and genome sequence analyses of Megasphaera sp. strain DISK18 from dental plaque of a healthy individual reveals commensal lifestyle. Sci Rep 2016;6(1):33665. https://doi.org/10.1038/srep33665

47.	Na HS, Jung N-Y, Song Y, et al. A distinctive subgingival microbiome in patients with periodontitis and Alzheimer’s disease compared with cognitively unimpaired periodontitis patients. J Clin Periodontol 2024;51(1):43–53. https://doi.org/10.1111/jcpe.13880

48.	Boopathy R, Robichaux M, LaFont D, Howell M. Activity of sulfate-reducing bacteria in human periodontal pocket. Can J Microbiol 2002;48(12):1099–1103. https://doi.org/10.1139/w02-104

49.	Hawkes CG, Hinson AN, Vashishta A, et al. Selenomonas sputigena Interactions with Gingival Epithelial Cells That Promote Inflammation. Infect Immun;91(2):e00319-22. https://doi.org/10.1128/iai.00319-22

50.	Downes J, Munson M, Wade WG. Dialister invisus sp. nov., isolated from the human oral cavity. Int J Syst Evol Microbiol 2003;53(Pt 6):1937–1940. https://doi.org/10.1099/ijs.0.02640-0

51.	Camelo-Castillo AJ, Mira A, Pico A, et al. Subgingival microbiota in health compared to periodontitis and the influence of smoking. Front Microbiol 2015;6:119. https://doi.org/10.3389/fmicb.2015.00119

52.	Lim YK, Park S-N, Lee W-P, et al. Lautropia dentalis sp. nov., Isolated from Human Dental Plaque of a Gingivitis Lesion. Curr Microbiol 2019;76(11):1369–1373. https://doi.org/10.1007/s00284-019-01761-1

53.	Downes J, Dewhirst FE, Tanner ACR, Wade WG. Description of Alloprevotella rava gen. nov., sp. nov., isolated from the human oral cavity, and reclassification of Prevotella tannerae Moore et al. 1994 as Alloprevotella tannerae gen. nov., comb. nov. Int J Syst Evol Microbiol 2013;63(Pt 4):1214–1218. https://doi.org/10.1099/ijs.0.041376-0

54.	Relvas M, Regueira-Iglesias A, Balsa-Castro C, et al. Relationship between dental and periodontal health status and the salivary microbiome: bacterial diversity, co-occurrence networks and predictive models. Sci Rep 2021;11:929. https://doi.org/10.1038/s41598-020-79875-x

55.	Becker MR, Paster BJ, Leys EJ, et al. Molecular analysis of bacterial species associated with childhood caries. J Clin Microbiol 2002;40(3):1001–1009. https://doi.org/10.1128/JCM.40.3.1001-1009.2002

56.	Cultivable Anaerobic Microbiota of Severe Early Childhood Caries | Journal of Clinical Microbiology. Available from: https://journals.asm.org/doi/10.1128/jcm.02427-10. Accessed August 31, 2026

57.	Rupf S, Merte K, Eschrich K, Kneist S. Streptococcus sobrinus in children and its influence on caries activity. Eur Arch Paediatr Dent Off J Eur Acad Paediatr Dent 2006;7(1):17–22. https://doi.org/10.1007/BF03320810

58.	Mantzourani M, Gilbert SC, Sulong HNH, et al. The isolation of bifidobacteria from occlusal carious lesions in children and adults. Caries Res 2009;43(4):308–313. https://doi.org/10.1159/000222659

59.	Cho H, Ren Z, Divaris K, et al. Selenomonas sputigena acts as a pathobiont mediating spatial structure and biofilm virulence in early childhood caries. Nat Commun 2023;14(1):2919. https://doi.org/10.1038/s41467-023-38346-3

60.	Siqueira Jr JF, Silva WO, Romeiro K, et al. Apical root canal microbiome associated with primary and posttreatment apical periodontitis: A systematic review. Int Endod J 2024;57(8):1043–1058. https://doi.org/10.1111/iej.14071

61.	Chen T, Marsh PD, Al-Hebshi NN. SMDI: An Index for Measuring Subgingival Microbial Dysbiosis. J Dent Res 2022;101(3):331–338. https://doi.org/10.1177/00220345211035775

62.	Socransky S s., Haffajee A d., Cugini M a., Smith C, Kent Jr. RL. Microbial complexes in subgingival plaque. J Clin Periodontol 1998;25(2):134–144. https://doi.org/10.1111/j.1600-051X.1998.tb02419.x

63.	Antezack A, Etchecopar-Etchart D, La Scola B, Monnet-Corti V. New putative periodontopathogens and periodontal health-associated species: A systematic review and meta-analysis. J Periodontal Res 2023;58(5):893–906. https://doi.org/10.1111/jre.13173

64.	Lafaurie GI, Castillo DM, Iniesta M, et al. Differential analysis of culturable and unculturable subgingival target microorganisms according to the stages of periodontitis. Clin Oral Investig 2023;27(6):3029–3043. https://doi.org/10.1007/s00784-023-04907-5
